# Data-driven risk assessment framework for railway operation accidents based on an improved Bayesian network

**DOI:** 10.1371/journal.pone.0361856

**Published:** 2026-11-06

**Authors:** Rui Yang, Xuewei Li, Guanyi Liu

**Affiliations:** 1 School of Economics and Management, Beijing Jiaotong University, Beijing, China; 2 Transportation and Economic Research Institute, China Academy of Railway Sciences Corporation Limited, Beijing, China; Kharkiv National Automobile and Highway University, UKRAINE

## Abstract

As a cornerstone national infrastructure, railway systems have faced increasingly intensive operational demands, escalating accident risks that threat socio-economic stability and public welfare. Therefore, ensuring operational safety is of paramount importance. This study develops an effective framework for assessing railway operational accident risks by integrating an improved Bayesian network. Firstly, to mitigate data imbalance, the Adaptive Synthetic Sampling algorithm is introduced to establish a reliable and representative dataset. Subsequently, the AcciMap theory is introduced to form a multi-level risk assessment system. Secondly, to address the epistemic uncertainty within unstructured data, the Bayesian network is combined with hesitant cloud model. Finally, the performance of the proposed model is validated through different methods. The results demonstrate that the proposed model scientifically identifies the critical risk influencing factors of railway operation and can provide reliable and accurate risk assessment results. This study provides valuable insights for railway operation decision-makers to develop reasonable accident prevention measures and valuable support for railway operation risk management.

## 1 Introduction

Railway transportation plays a vital role in the national economies, offering an unparalleled cost advantage for passenger and cargo transportation. However, the rapid growth in travel demand and freight circulation has led to continuous expansion of network density and increasing train operating speeds, posing substantial challenges to railway operational safety [1]. According to statistics from the National Railway Administration of China, approximately 8,000 railway operational accidents occurred nationwide between 2016 and 2024, resulting in roughly 6,000 deaths. Similarly, data from the U.S. Federal Railroad Administration (FRA) indicate that nearly 110,000 railway accidents took place in the United States from 2013 to 2022, claiming close to 8,000 lives. These accidents have caused enormous economic losses and pose severe threats to passenger safety [2]. Consequently, a thorough understanding of the root causes of such accidents is of critical practical significance for strengthening railway operational safety management.

Railway accidents are inherently marked by stochastic uncertainties and complexities. Therefore, identifying the latent risk influence factors (RIFs) responsible for these accidents is pivotal to enhancing train operation safety [3]. This means that it is necessary to ensure the high quality of accident reports, as report content must include detailed information. Nevertheless, data collection is frequently constrained by issues such as incomplete records, inconsistent data formats, and other limitations [4]. However, various limitations often arise during the data collection process, such as incomplete data and inconsistent data structures. In addition, due to the complex coupling effects of different types of factors involved in railway accidents, obtaining high-quality data usually requires a significant amount of resource investment. To address this issue, a number of literature leverages expert elicitation and subjective heuristics to investigate railway operation risks from a primarily qualitative perspective [5].

However, such studies are dependent on subjective expert experience, lacking sufficient support from objective historical data. Under the complex interactions among RIFs, the accuracy of expert judgments is increasingly challenged. Therefore, researchers have begun to adopt quantitative methods, including probabilistic risk assessment, Monte Carlo simulation, and risk matrices. Among advanced techniques for railway accident risk analysis, Bayesian networks (BNs) stand out as a powerful tool for representing and reasoning under uncertainty. Owing to their ability to effectively handle uncertainty and complex causal relationships, BNs have been widely applied in reliability and risk analysis across various domains [6,7].

Despite their strengths, BNs are limited by their strong dependence on high-quality and complete datasets. In cases of data scarcity or inaccuracy, the precision of BN inferences can be significantly compromised, potentially leading to inconsistent, biased, or erroneous results. When foundational or complete data are unavailable, expert knowledge often becomes the key source of support for railway accident modeling [8]. However, expert knowledge is inherently subjective, carrying intrinsic uncertainty, vagueness, and variability. This subjectivity can amplify errors in risk assessment. Although some scholars have applied Dempster–Shafer (DS) evidence theory to aggregate evaluations from multiple experts in an effort to reduce assessment errors [9], the present study finds that unstructured data derived from expert experience remain limited in quantity, heavily laden with subjective judgment, and strongly influenced by factors such as idiosyncratic cognitive predispositions, systemic intricacies, and semantic vagueness. These characteristics result in data that exhibit epistemic hesitation and inherent uncertainty.

Incorporating hesitation into BNs poses a significant challenge, as conventional BNs possess limited innate capacity to directly parse vacillating linguistic feedback. In complex risk scenarios where expert opinions diverge significantly, the algorithmic burden of BN inference escalates rapidly, potentially undermining computational tractability and precipitating erratic or spurious outcomes [10]. To address this limitation, the present study proposes an innovative enhancement of the BN by integrating the hesitant cloud model. This approach more effectively captures and processes the fuzziness and uncertainty embedded in expert opinions, enabling the aggregation of multiple expert judgments to derive robust prior probabilities for subjective nodes, thereby substantially reducing errors induced by subjective data.

In summary, we develop a data-driven framework for railway accident risk assessment. The framework aims to systematically analyze historical railway accident data, identify key RIFs, and provide practical solutions for risk mitigation. The main contributions are as follows:

(1) This study investigates the issue of data imbalance in railway accident reports. By leveraging the Adaptive Synthetic Sampling (ADASYN) algorithm, the proposed framework ensures the validity of the data. The data preprocessing establishes a rigorous computational substrate for the robust development and empirical validation of subsequent risk assessment.
(2) To overcome the limitations of fragmented risk identification and the structural ambiguity, this study innovatively integrates the AcciMap theory with accident reports to construct a comprehensive railway operational accident risk assessment system. Unlike conventional single-dimensional models (e.g., human–machine–environment–management frameworks), the proposed multi-level indicator system identifies risk factors and their interactions, offering a holistic and scientifically grounded basis for risk assessment and prediction.
(3) To tackle the challenge of incorporating unstructured data and handling high levels of hesitation and uncertainty in multi-expert opinions within BN models, this study integrates the hesitant cloud model to enhance the BN. By aggregating expert judgments to simulate prior probabilities of subjective nodes, the approach significantly mitigates errors arising from subjective data.
(4) This study proposes different methods for identifying critical RIFs, including mutual information, weighting influence analysis, and scenario analysis, to conducts a rigorous sensitivity appraisal of railway operational risks. This integrated approach transcends the inherent constraints of conventional risk assessment paradigms through the quantitative dissection of individual factor contributions. Consequently, it achieves a higher degree of precision in quantifying the dynamic sensitivities of key RIFs and elucidating their evolutionary trajectories across diverse operational contexts.

The remainder of this paper is organized as follows: Section 2 reviews relevant literature. Section 3 presents the proposed railway operational accident risk assessment framework, encompassing data preprocessing, system construction, BN modeling, hesitant cloud model integration, model validation, and sensitivity analysis. Section 4 demonstrates the practical performance of the framework through a case study. Section 5 discusses the discussion and implications, and Section 6 concludes the paper and outlines directions for future research.

## 2 Literature review

### 2.1 Risk analysis of railway operational accidents

Railway operational accidents are characterized by sudden onset, high complexity, and significant uncertainty, with various risk factors interacting in nonlinear ways that amplify overall risk. Consequently, numerous scholars have investigated this problem using diverse methodologies. For instance, Liu et al. [11] proposed a knowledge graph-based approach for handling railway operational accidents, employing graph embedding and topological analysis to predict latent operational risks and thereby enhance risk management capabilities. Dindar et al. [12] utilized historical accident datasets and applied Bayesian inference, decision trees, and Petri nets to benchmark railway safety performance, offering valuable insights into operational reliability. Liu et al. [13] analyzed the pathways between latent and active errors in 407 Chinese railway accident reports, identifying key contributing factors through causal modeling. Liu et al. [14] applied association rule mining to identify critical risk factors at hazardous locations of rural road traffic accidents. Dindar et al. [15] developed a knowledge graph-based framework for railway operational accident management, integrating the N-K model with functional resonance analysis to quantify risk variability. Liu et al. [16] employed the N-K and system dynamics (SD) models to investigate coupling mechanisms of safety accident risks in hydrogen energy systems, providing useful references for railway-related energy hazards. Liu et al. [17] constructed an analytical framework for operational risk coupling in maritime autonomous surface ship (MASS) navigation scenarios using the Functional Resonance Analysis Method (FRAM), which holds implications for railway automation risk analysis. Li et al. [18] proposed an approach to the emergence and prevention of high-speed railway accident risks from the perspective of resilience engineering, identifying critical factors throughout the accident process. Liu et al. [19] conducted a systems-based risk analysis of tram operations, combining Monte Carlo simulation with functional resonance analysis, demonstrating improved safety performance in urban rail systems. Wang et al. [20] developed a coupling risk assessment method for metro construction, using a weighted Bayesian network for quantitative evaluation. Liu et al. [21] evaluated coupling links in the maritime transport of hazardous chemicals based on the FRAM model, offering transferable insights for railway dangerous goods transportation. Song et al. [22] integrated a BBN-NK-Boltzmann model to analyze the network risk evolution of tunnel fires, exploring complex risk interactions in confined railway spaces.

In summary, Bayesian networks have gained widespread popularity due to their comprehensive modeling capability. By representing causal relationships among variables through directed acyclic graphs, they offer strong adaptability and interpretability in the analysis of complex systems. For these reasons, the present study adopts Bayesian networks as the core modeling approach for railway operational accident risk.

### 2.2 Applications of BN in risk analysis

Bayesian networks provide a probabilistic graphical modeling framework that effectively handles uncertainty and complex causal relationships, and have been extensively applied in railway accident risk analysis. They can intuitively represent causal dependencies between variables, process uncertain information, and support both risk prediction and causal diagnosis. Accordingly, researchers have explored their application from various perspectives. Liu et al. [23] developed a data-driven Bayesian network for causal analysis of freight railway accidents under specific scenarios, effectively capturing conditional dependencies among accident causes across different operational contexts. Wu et al. [24] integrated knowledge-element theory with dynamic Bayesian networks to construct a multi-scenario deductive analysis framework for railway emergency events, enabling real-time risk inference and decision optimization. Li et al. [25] proposed a weighted Bayesian network approach for structural safety assessment of high-speed railway sections, accounting for multi-factor coupling effects and ranking factor importance. Dindar et al. [5] established a hierarchical Bayesian model to investigate the impact of extreme weather conditions on railway switch component failures, revealing dynamic patterns of derailment risk through posterior inference. Liang et al. [26] conducted risk analysis of railway level-crossing accidents using a causal Bayesian network, incorporating structural constraints to improve model rationality and explanatory power. Feng et al. [27] built a Bayesian network-based risk assessment model for evaluating operational risks of China-Europe freight trains under the Belt and Road Initiative, with a focus on identifying key risk factors in cross-border transportation. Zhang et al. [28] developed a Bayesian network scenario inference model for inland intelligent ship collision accidents, demonstrating risk propagation pathways under different scenarios. Uflaz et al. [29] combined evidence theory with rule-based Bayesian networks to quantify cyber-attack risks in transportation networks, providing a reference for railway signaling and communication system security assessment. Deng et al. [30] proposed a probabilistic network approach to quantify the risk reduction effects of safety barriers and optimize barrier configuration strategies via Bayesian inference. Bauranov et al. [31] utilized Bayesian networks to evaluate the impact of introducing new technologies on collision risks in transportation systems, offering insights for safety assessment during railway intelligent transformation. Qu et al. [32] developed a multi-source data-driven Bayesian network framework, employing data fusion and uncertainty handling techniques to construct datasets for high-seas maritime accident risk analysis. Fan et al. [33] proposed a dynamic Bayesian network risk assessment framework to address uncertainty factors in shipping routes, effectively identifying critical risk-influencing factors.

Overall, existing studies have enhanced Bayesian network performance by integrating machine learning, data-driven approaches, and intelligent algorithms. However, most research overlooks the critical role of data quality in Bayesian network modeling. For unstructured nodes within the network, reliance on expert evaluation remains common. Yet the inherent subjectivity and hesitation of experts introduce uncertainty into the data. Cloud models offer an effective means to address fuzzy information by mapping qualitative concepts to quantitative descriptions. Therefore, the present study employs cloud models to mitigate errors arising from expert evaluation.

### 2.3 Applications of cloud models in risk assessment

As a mathematical model of uncertainty, the cloud model effectively integrates randomness and fuzziness. It has been widely applied in risk analysis to handle uncertain information and multi-source data in complex systems, providing quantitative risk evaluation and decision support. The advantages of cloud models in processing unstructured data are now well recognized, with applications in metro, aviation, and maritime transportation domains. For example, Xu et al. [34] developed a cloud model-enhanced framework for risk assessment of technical innovation in railway engineering, combining composite weighting methods to construct cloud diagrams and analyze multidimensional risk factors in complex regions. Zhang et al. [35] constructed a cloud-based diagnostic model for evaluating metro emergency response capability, leveraging 4R crisis management theory to handle uncertain information and enable timely response to emergencies. Wang et al. [36] introduced the cloud model for urban rail transit operational safety assessment, accounting for uncertainty in key influencing factors and employing an S-shaped utility function to evaluate risk levels. Deng et al. [37] proposed a cloud model-assisted risk assessment method for aviation unsafe events, using QAR data to quantify risk grades. Li et al. [38] developed a cloud model-based safety assessment approach for autonomous vehicle decision-making, evaluating real-time risk through cloud parameters such as expectation and entropy. Yang et al. [39] integrated Bayesian networks with cloud models for probabilistic risk assessment of maritime collision accidents, effectively handling multi-source uncertainty. Sun et al. [40] proposed a cloud model-based method for assessing pedestrian-vehicle conflict risks, using a hierarchical grey entropy cloud model to classify risks and identify critical risk factor combinations in conflict scenarios. Chen et al. [41] constructed a cloud model-based framework for debris flow disaster risk assessment, improving boundary coverage through Monte Carlo simulation to support fuzzy decision-making. Wu et al. [42] developed a cloud model-based approach for assessing risks to existing tunnels during tunnel construction, quantifying the impact of uncertain factors. Li et al. [43] introduced an improved normal swing hesitant fuzzy FMEA model for complex system risk assessment, incorporating hesitant clouds to handle expert uncertainty and improve prediction accuracy. Wang et al. [44] proposed a cloud model-based navigation safety risk assessment system for coast guard vessels, constructing a hierarchical indicator system to support risk prediction. Zhang et al. [45] developed a cloud model-enhanced method for assessing collapse risks in underground foundation pits, fusing evidence theory to process multi-source data and reduce subjectivity in expert evaluation.

In summary, cloud models provide an effective tool for managing information fuzziness and randomness in risk assessment. However, they primarily focus on expressing uncertainty for individual indicators. In practice, experts often exhibit hesitation when evaluating a given indicator. Hesitant fuzzy sets, as an extension of fuzzy sets, better capture the hesitancy inherent in evaluation data. Therefore, this study integrates hesitant fuzzy sets with cloud models to achieve a more comprehensive treatment of uncertainty and hesitation in complex decision-making processes.

### 2.4 Differences from existing literature

Based on the limitations of existing research methods, the main contributions of this study are as follows:

(1) Bayesian network is a classic model used to analyze railway accidents. However, the inference results of Bayesian networks depend on data quality. Previous studies have overlooked the imbalanced characteristics of railway accident data, resulting in biased inference results [13,26]. Therefore, the framework of this study introduces the ADASYN algorithm to balance the dataset, ensuring that the model can accurately predict low probability but high severity accident risks.
(2) Traditional railway risk assessment models typically analyze risk factors in a single dimensional category, such as people, machines, environment, and management [28,29]. However, it is not common to analyze the impact of government and enterprises on railway accidents from a macro perspective. This study constructs a novel multi-level research framework by introducing the AcciMap theory. This enables the constructed BN model to not only capture individual factors, but also the causal relationship between management errors and physical accidents.
(3) In railway risk assessment, many key risk factors are qualitative and require expert evaluation. Traditional cloud models [39] or fuzzy methods [42] can simulate the fuzziness of a single evaluation, but they can be difficult to model when multiple experts have disagreements or hesitations between multiple choices. The hesitant cloud model proposed in this study effectively combines hesitant fuzzy sets with cloud models. It allows multiple experts to provide conflicting linguistic judgments, successfully aggregating their opinions into reliable prior probabilities and reducing subjective biases.

In summary, our framework integrates these methods and provides a more complete, accurate, and reliable tool for railway operation risk management. Table 1 summarizes the comparison between our proposed framework and recent representative methods.

**Table 1:** Comparison between the proposed method and previous research methods.

Model	Captures Macro-level Structural Causality	Inter-expert Cognitive Hesitation	Models Micro-level Stochastic Fuzziness	Mitigates Empirical Data Imbalance
Standard Causal BN [13,26]	Yes	No	No	No
Cloud-enhanced BN [39]	Yes	No	Yes	No
DS-Evidence BN [28,29]	Yes	Yes	No	No
Hesitant Fuzzy FMEA [42]	No	Yes	Yes	No
Proposed Framework	Yes	Yes	Yes	Yes

## 3 Methodology

This study adopts a data-driven approach to construct a risk assessment model for railway operational accidents, with the aim of providing an effective framework for risk management. The application of BNs requires the integration of input data. However, accident reports are typically unstructured, and the identification of risk nodes often relies heavily on expert judgment. Such data are characterized by incompleteness, knowledge uncertainty, and judgmental ambiguity. To address these issues, this study innovatively integrates the strengths of hesitant fuzzy sets and cloud models to develop a hesitant cloud model, which effectively aggregates and simulates prior probability judgments from multiple experts.

The overall research framework is illustrated in Fig 1. First, an initial railway operational accident database is established using officially published accident reports and reliable data sources. The ADASYN algorithm is applied to address the class imbalance problem. Second, drawing on an extensive review of existing literature and accident reports, a railway operational accident risk indicator system is constructed based on the AcciMap approach. Both quantitative and qualitative methods are used to determine the state classifications of RIFs, thereby building the BN model for railway operational accident risk. Thirdly, the proposed hesitant cloud model is employed to derive prior probabilities of parent nodes, while gradient descent is used to obtain the conditional probability tables among RIFs. In the fourth stage, the model’s performance is validated through multiple evaluation methods. Finally, mutual information, weighted influence analysis and scenario analysis are utilized to comprehensively identify and analyze the key factors influencing railway operational accident risk.

**Fig 1:**
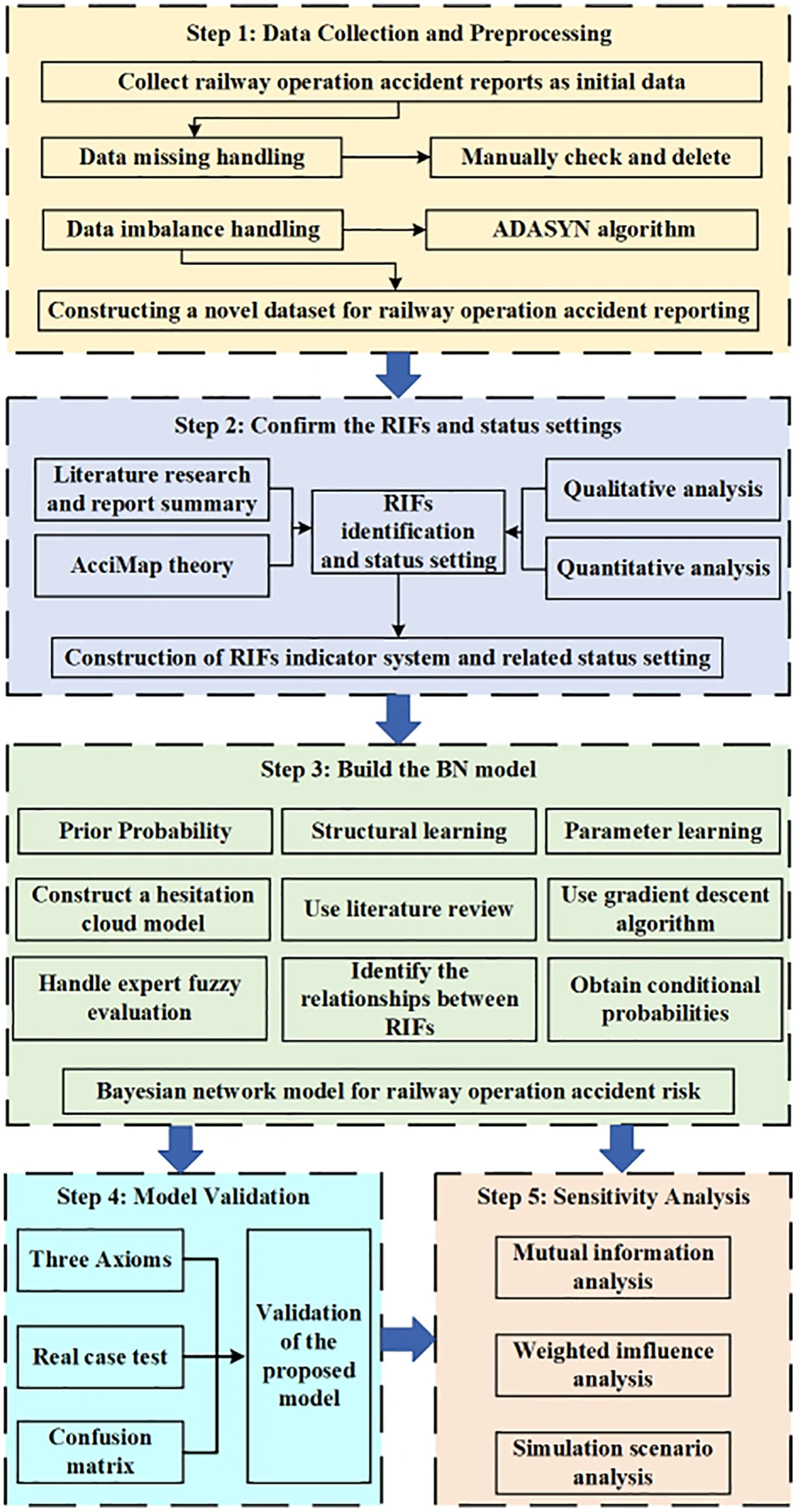
Research framework for risk assessment of railway operation accidents.

### 3.1 Data preprocessing

The preliminary database comprises a large volume of accident documents, providing basic information such as accident time, location, and causes. In addition, the reports contain in-depth analyses of accident scenes, casualty statistics, economic losses, and penalty decisions. By examining and enhancing the foundational data embedded in these reports, this study constructs a basic railway accident database to build BN model.

Among the collected accident reports, the level of detail in accident descriptions varies. Some reports fail to fully describe the accident process, rendering them insufficient for extracting valid information. Therefore, each report is carefully reviewed, and incomplete reports are excluded [46]. Following this screening, accident data are identified and incomplete or anomalous entries are corrected. It is observed that certain risk categories exhibit significant quantitative disparities compared to others. Such imbalance may cause the model to prioritize majority classes, thereby impairing its ability to accurately detect minority-class instances. To mitigate this challenge, the ADASYN algorithm is employed to handle imbalanced data [47]. The detailed steps of the algorithm are as follows:

Step 1. Assume the training set D contains N samples, where $X_{i}=\left(X_{\text{1}},X_{\text{1}},\ldots X_{n}\right)$ represents the sample set and $Y_{i}=\left(Y_{\text{1}},Y_{\text{1}},\ldots Y_{m}\right)$ the class label set. Let $N_{l}$ and $N_{l}$ denote the number of majority-class and minority-class samples, respectively, with $N_{s}\leq N_{l},N_{s}{+N}_{l}=N$. The imbalance ratio is thus:

$$\begin{array}{c} \begin{array}{c} d=\frac{N_{s}}{N_{l}} \end{array} \end{array}$$ (1)

Step 2. To balance the entire dataset, the total number of synthetic samples to be generated for the minority class is calculated as:

$$\begin{array}{c} \begin{array}{c} G=\left(N_{l}-N_{s}\right)\times \beta \end{array} \end{array}$$ (2)

where the parameter β represents the desired balance degree after generating synthetic samples.
Step 3. For each minority sample, the K nearest neighbors are identified based on Euclidean distance, and the ratio $R_{i}$ is computed as:

$$\begin{array}{c} \begin{array}{c} R_{i}=\frac{\Delta i}{K} \end{array} \end{array}$$ (3)

where Δi represents the number of majority-class samples among the K nearest neighbors of the $X_{i}$ minority sample.
Step 4. Normalize the ratio:

$$\begin{array}{c} \begin{array}{c} \hat{R_{i}}=\frac{R_{i}}{\sum_{i=\text{1}}^{N_{s}}R_{i}} \end{array} \end{array}$$ (4)

Step 5. Determine the number of synthetic samples to be generated for each minority sample:

$$\begin{array}{c} \begin{array}{c} N_{i}=\hat{R_{i}}\times G \end{array} \end{array}$$ (5)

Step 6. Generate synthetic data for each minority sample:

$$\begin{array}{c} \begin{array}{c} S_{i}=X_{i}+\left(X_{random}-X_{i}\right)\times \alpha \end{array} \end{array}$$ (6)

where α is a random number uniformly distributed between 0 and 1.

It is worth noting that this study uses the ADASYN algorithm to achieve data smoothing and distribution balance in handling imbalanced data classes. In the AcciMap framework, RIF is a discrete metric, and before vector synthesis, the classification state is first encoded as a discrete integer, e.g., S1 = 1，S2 = 2. To ensure that all synthesized data is meaningful, the nearest integer rounding rule is strictly enforced immediately after the online generation step. This ensures that the score values are automatically mapped back to their nearest valid discrete category, successfully preserving the physical meaning of each generated risk scenario.

### 3.2 RIFs identification

To systematically parse the synthesized corpus of railway incident records, this study utilizes the AcciMap theory to extract the critical RIFs. AcciMap theory, proposed by Rasmussen, is a systemic model for understanding accident causation [48], as conceptually illustrated in Fig 2. This framework facilitates a multi-dimensional deconstruction of accident etiology, transcending linear causality by examining the complex interplay between disparate sociotechnical strata.

**Fig 2:**
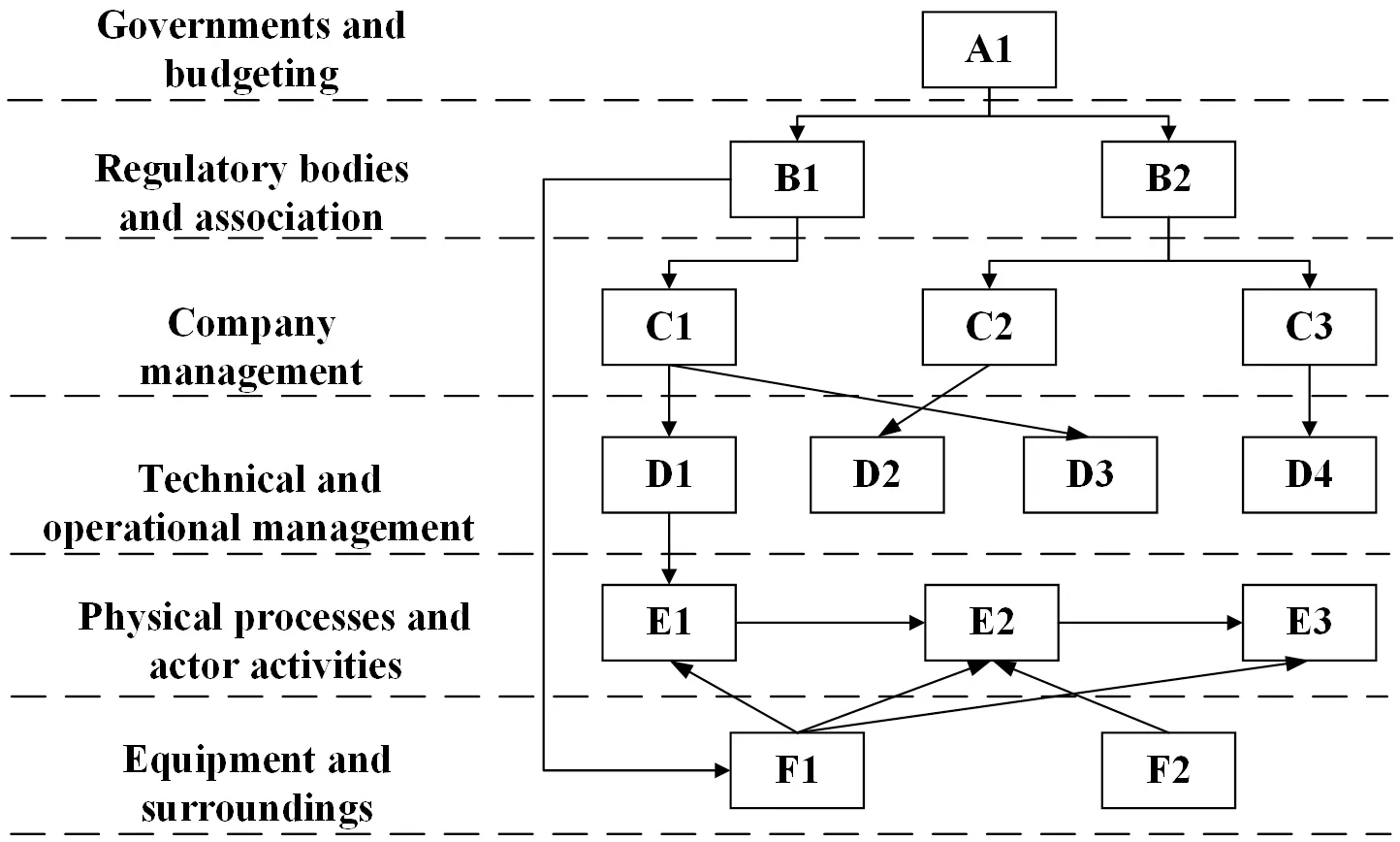
Conceptual diagram of the AcciMap framework.

Specifically, the framework encompasses government and budgeting, regulatory bodies and association, company management, technical and operational management, physical processes and actor activities, as well as equipment and surroundings [49]. A brief explanation is as follows.

(1) Government and budgeting reflect overall national safety philosophies, local governance responsibilities, safety regulations, and fiscal allocations for safety initiatives.
(2) Regulatory bodies and associations focus on risk factors originating from emergency management agencies, market regulators, and similar entities.
(3) Company management, combined with technical and operational management, examines risk factors related to safety management systems, organizational coordination, and on-site safety decision-making.
(4) Physical processes and actor activities assess unsafe acts, decisions, tasks, and actions of involved individuals, influenced collectively by factors at various levels.
(5) Equipment and environmental factors describe risks associated with system-related equipment and the surroundings.

Drawing upon the hierarchical tenets of Rasmussen’s sociotechnical paradigm, we identify relevant risk factors and adapt the AcciMap structure into a customized six-dimension indicator system，including government regulation, enterprise management, operation and maintenance management, risk inspection, physical processes and actor activities, and equipment and surroundings. While conventional safety taxonomies often categorize railway accidents types (e.g., collisions, derailments, or explosions), the present study embraces a causal perspective. This shift in perspective ensures high congruence with empirical railway safety, where the excavation of systemic latent precursors—such as policy lapses, regulatory gaps, and human errors—is accorded higher diagnostic primacy than mere surface-level accident classification. Consequently, an exhaustive synthesis of extant literature and historical accident reports are performed, yielding 25 key indicators that represent the primary RIFs, as delineated in Fig 3.

**Fig 3:**
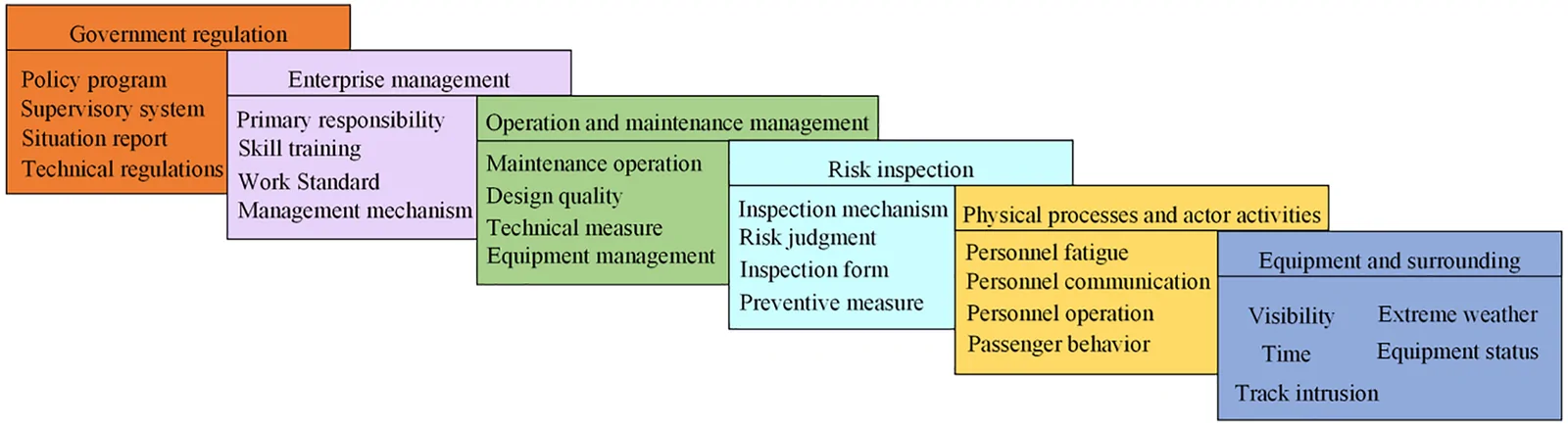
RIFs of railway operation accidents based on AcciMap theory.

### 3.3 Hesitant cloud model

A major challenge in modeling nodes within BN is that sample data supporting the nodes are not always structured; unstructured data, predominantly derived from qualitative expert judgments and small samples, are common [50]. Such non-structured judgments are frequently influenced by subjective factors, including individual knowledge biases, environmental complexity, and information ambiguity stemming from evaluator hesitation, resulting in substantial uncertainty during parameter learning.

Conventional BN exhibit limitations when processing unstructured data. These data are limited in quantity and heavily influenced by subjective judgments affected by expert bias, environmental variability, and fuzzy information, leading to inherent fuzziness and uncertainty [51]. Traditional methods typically rely on crisp, quantitative inputs and thus struggle to effectively capture and process such fuzziness and evaluator hesitation, making them susceptible to subjective bias during parameter learning and resulting in reduced predictive accuracy and model stability.

To overcome this limitation, this study proposes the use of a hesitant cloud model to objectively model the prior probabilities of subjective nodes by aggregating opinions from multiple experts. Leveraging the unique strengths of the hesitant cloud model, this approach effectively handles data fuzziness and evaluator hesitation, thereby providing more accurate and reliable parameter estimates. This method successfully minimizes errors induced by subjective data and enhances the overall accuracy and stability of the BN model.

#### 3.3.1 Cloud model.

Proposed by Deyi Li, the cloud model constitutes a stochastic-fuzzy synthesis designed to bridge the gap between qualitative linguistic concepts and their quantitative counterparts [52]. It synergistically encapsulates imprecision and stochasticity, effectively mapping the dual-uncertainty inherent in human knowledge through three pivotal numerical descriptors: Expectation (Ex), Entropy (En), and Hyper-entropy (He).

(1) Expectation (Ex) denotes the cloud’s focal coordinates within the universe of discourse, signifying the quintessential value that anchors the qualitative concept and serves as its most objective representation.
(2) Entropy (En) quantifies the conceptual granularity and distributional variance, reflecting the elasticity of the linguistic boundary and the range of values acceptable within that concept.
(3) Hyper-entropy (He) assesses the secondary uncertainty (the uncertainty of entropy), characterizing the structural stability and cohesion of the cloud droplets [53].

The cloud model consists of two primary algorithms: the forward cloud generator (transforming qualitative concepts into quantitative data) and the backward cloud generator (transforming quantitative data into qualitative concepts).

(1) Forward cloud generator: Using the three characteristic values (Ex, En, He), it generates normally distributed random values to produce cloud drops that collectively reflect the distribution of the qualitative concept.
(2) Backward cloud generator: It analyzes a set of quantitative data to compute the corresponding Ex, En, and He, thereby deriving the qualitative concept represented by the data.

In risk assessment, qualitative information must be processed and quantified. This study relies on expert evaluations of various indicators. The forward cloud generator converts qualitative comments into numerical cloud characteristics, which are then aggregated at the corresponding parent nodes using virtual cloud algorithms. This process captures both the uncertainty inherent in expert judgments and the uncertainty introduced by the algorithm itself.

#### 3.3.2 Hesitant fuzzy set.

Hesitant fuzzy set (HFS) theory is an advanced extension of fuzzy set theory [54]. Fuzzy set theory introduced the concept of membership degrees to effectively describe vagueness and uncertainty in the real world.

Nevertheless, in the intricate decision-making environment, due to incomplete or imprecise information and the inherent hesitation of decision-makers, an element may be associated with multiple possible membership degrees. Hesitant fuzzy sets were developed to more accurately capture this hesitancy and uncertainty. The formal definition is as follows:


$$\begin{array}{c} \begin{array}{c} D=\left\{\left\langle x,h(x)\right\rangle |x\in X\right\} \end{array} \end{array}$$ (7)

where X denotes a fixed set. h(x) represents the hesitant fuzzy element (HFE)—a discrete set of potential values within the interval [0,1] provided by experts. To synthesize these multi-valued inputs into a cohesive metric, the Hesitant Fuzzy Weighted Average (HFWA) operator is employed:


$$\begin{array}{c} \begin{array}{c} HFWA\left(h_{\text{1}},h_{\text{2}}{,}^{\cdots },h_{n}\right)={⊕}_{i=\text{1}}^{n}w_{i}h_{i}=\overset{n}{\underset{\gamma \text{1}\in h\text{1},\gamma \text{2}\in h\text{2},\cdots ,\gamma n\in hn}{⋃}}\left\{\text{1}-\prod_{i=\text{1}}^{n}{\left(\text{1}-\gamma_{i}\right)}^{w_{i}}\right\} \end{array} \end{array}$$ (8)

where $w_{i}$ represents the weight of the i−th HFE, satisfying the normalization constraint $\sum_{i=\text{1}}^{n}\;w_{i}=\text{1}$. By permitting an element to be associated with multiple membership values, the HFS framework distills the underlying epistemic uncertainty inherent in expert elicitation [55]. This approach not only extends the theoretical reach of fuzzy sets but also provides a more comprehensive information for subsequent risk inference, significantly bolstering the reliability of safety critical decisions in railway operations.

To improve the precision of the risk assessment, we prioritize mitigating biases inherent in heuristic-based expert elicitation. Collective expert insights are synthesized to ensure a multi-faceted characterization of the risk landscape, effectively neutralizing individual cognitive outliers. The numerical ensembles generated through cloud-transformed linguistic inputs are conceptualized as generalized hesitant fuzzy sets. Consequently, the hesitant cloud model is deployed to fuse these disparate cloud droplets, obtaining the final integrated assessment result.

#### 3.3.3 Construction of the hesitant cloud model.

The construction steps of the Hesitation Cloud model are as follows:

Step 1. Construct a set of evaluation comments for all indicators of the assessment object.
The evaluator provides comments on all indicators of the evaluated object $\{A_{i}\},i=\text{1},\text{2},\cdots ,m$, where i is the i−th indicator of the evaluated object. If there are n evaluators in total, the sub-comment matrix is $\left\{A_{\text{1}i},A_{\text{2}i}\cdot \cdot \cdot ,A_{ni}\right\}$
Step 2. Convert the comment set into cloud model numerical characteristic sets.
For single-sided constraint comments, the half-decreasing or half-increasing cloud transformation is applied Eq. 9. For double-sided constraint comments, Eq. 10 is used. This yields the sub-hesitant fuzzy matrices for Ex, En, He, respectively.

$$\begin{array}{c} \begin{array}{c} \left\{ \begin{array}{cc} & Ex=a\text{ or }b\\ & En=(b-a)/\text{3}\\ & He=k \end{array}\right.\; \end{array} \end{array}$$ (9)

$$\begin{array}{c} \begin{array}{c} \;\left\{ \begin{array}{cc} & Ex=(a+b)/\text{2}\\ & En=(b-a)/\text{6}\\ & He=k \end{array}\right.\; \end{array} \end{array}$$ (10)

where k is a constant with k=0.5. Specifically, the value of k is referenced to Zhang et al. [56].
Step 3. Generate hesitant cloud groups.
According to hesitant fuzzy theory, each numerical characteristic generates t hesitant fuzzy values, where $t=\prod_{i=\text{1}}^{n}\;l_{i}$. $l_{i}$ is the i−th evaluator. Forming a hesitant cloud group for the evaluation objects ${\{C_{\text{1}}(Ex_{\text{1}},En_{\text{1}},He_{\text{1}}),C}_{\text{2}}(Ex_{\text{2}},En_{\text{2}},He_{\text{2}}),\cdots ,C_{t}(Ex_{t},En_{t},He_{t})\}$.
Step 4. Compute the average hesitant cloud.
The fuzzy weighted average of hesitation for Ex, En, and He generates the average hesitation cloud for all indicators: $\{C_{i}(Ex_{i},En_{i},He_{i})\},i=\text{1},\text{2},...,m$
Step 5. Compute the comprehensive cloud.
Using the virtual cloud synthesis method, the hesitant clouds of individual indicators are aggregated to obtain the final synthesized comment cloud that preserves all evaluators’ information. The weighted virtual synthesis algorithm is given as follows:

$$\begin{array}{c} \begin{array}{c} \left\{ \begin{array}{c} @lcEx=\frac{Ex_{\text{1}}\times En_{\text{1}}\times w_{\text{1}}+Ex_{\text{2}}\times En_{\text{2}}\times w_{\text{2}}+\cdots +Ex_{n}\times En_{n}\times w_{n}}{En_{\text{1}}\times w_{\text{1}}+En_{\text{2}}\times w_{\text{2}}+\cdots +En_{n}\times w_{n}}\\ \;\;\;\;En=n_{\text{1}}\times w_{\text{1}}+En_{\text{2}}\times w_{\text{2}}+\cdots +En_{n}\times w_{n}\\ He=\frac{He_{\text{1}}\times En_{\text{1}}\times w_{\text{1}}+He_{\text{2}}\times En_{\text{2}}\times w_{\text{2}}+\cdots +Ele_{n}\times En_{n}\times w_{n}}{En_{\text{1}}\times w_{\text{1}}+En_{\text{2}}\times w_{\text{2}}+\cdots +En_{n}\times w_{n}} \end{array}\right. \end{array} \end{array}$$ (11)

Step 6. Map numerical characteristics of the cloud model to evaluation comments.

The forward normal cloud generator produces a cloud diagram representing the integrated evaluation concept. Depending on the purpose and required precision of the assessment, appropriate concept cloud drops are selected, and their distribution across defined evaluation intervals is observed to derive the final result. Specifically, evaluation intervals are predefined, and a number of cloud drops are generated using the cloud characteristics (Ex, En, He). The drops are classified into intervals, and the percentage of drops in each interval is calculated. The interval with the highest percentage determines the final mapped evaluation level.

### 3.4 Construction of the BN

#### 3.4.1 Bayesian network.

BN can be visualized as a directed acyclic graph (DAG) and mathematically expressed through the joint probability distribution over its variables, which may be discrete or continuous. A directed edge between two nodes carries a conditional probability that quantifies the strength of their causal relationship. When new evidence is entered into one or more nodes, Bayesian inference propagates through the network to update the posterior probabilities of the remaining nodes [57].

Assuming a set of discrete variables $\text{X}=\{X_{\text{1}},X_{\text{2}},\ldots ,X_{N}\}$, where $x_{i}$ represents a node state of $X_{i}$ and N represents the number of nodes. The joint probability calculation formula for BN is:


$$\begin{array}{c} \begin{array}{c} P(\text{X})=\prod_{i=\text{1}}^{N}\;P\left(x_{i}|x_{\text{1}},x_{\text{2}}\ldots x_{i-\text{1}}\right)-\prod_{i=\text{1}}^{N}\;P\left(x_{i}|pa\left(x_{i}\right)\right) \end{array} \end{array}$$ (12)

where $pa\left(x_{i}\right)=\left\{x_{\text{1}},x_{\text{2}}\ldots x_{i-\text{1}}\right\}$ represents the node state of all parent nodes of $X_{i}$. The posterior probability of estimating $X_{i}$ through Bayesian inference is as follows:


$$\begin{array}{c} \begin{array}{c} P\left(x_{i}|e\right)=P\left(x_{i}|e^{pa}\right)\cdot P\left(e^{ch}|x\right) \end{array} \end{array}$$ (13)

where e represents evidence input to one or more network nodes. The node receiving evidence can be the parent or child node of $x_{i}$. These nodes are marked as $e^{\text{pa}}$ and $e^{\text{ch}}$ respectively.

#### 3.4.2 Structure learning.

The directed graph is constructed to represent the topological relationships among RIFs. Connections between parent and child nodes indicate dependency or causal links. BN structure learning typically combines expert knowledge, machine learning, or both [58]. Relying solely on expert judgment may introduce subjectivity. Therefore, this study derives relationships among factors through literature review and expert brainstorming, as illustrated in Fig 4.

**Fig 4:**
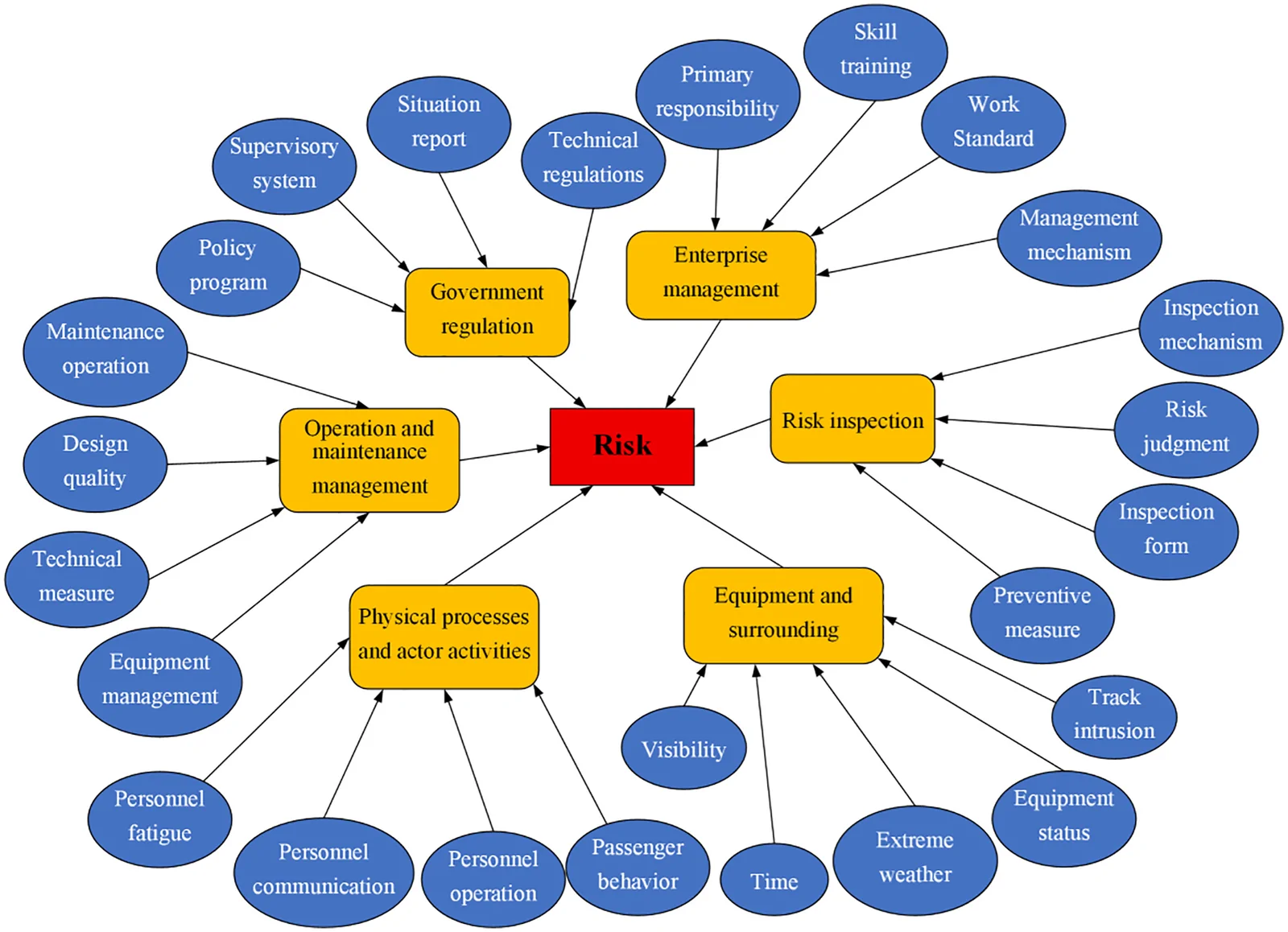
BN structure of railway operation accident risk.

#### 3.4.3 Parameter learning.

Following the structural formalization of the BN, it is imperative to quantify the conditional dependencies among nodes using the synthesized dataset. Conventional methodologies for node parameterization encompass Maximum Likelihood Estimation (MLE), Bayesian Estimation (BE), and the Expectation Maximization (EM) algorithm [13]. While EM-based heuristics excel in managing latent constructs or data sparsity, the dataset in this study is relatively complete. Furthermore, although MLE offers a streamlined approach, it is frequently susceptible to localized convergence traps, especially when initialized within convoluted risk landscapes. Meanwhile, the BE involves intensive posterior characterization, which may impose substantial computational overhead in high-dimensional railway safety systems.

Consequently, this study leverages a gradient descent-based optimization strategy for parameter estimation. This algorithm facilitates the integration of advanced optimization methods, thereby accelerating convergence rates and enhancing algorithmic flexibility.

In BNs, given historical data e from existing databases, the conditional probability distribution of a specific variable V under evidence conditions can be expressed as $x=P\left(V|e\right)=\left(x_{\text{1}},x_{\text{2}},\ldots ,x_{n}\right)$. Meanwhile, for its conditional probability P(V|e), there exists a prior assignment $y=\left(y_{\text{1}},y_{\text{2}},\ldots ,y_{n}\right)$, which reflects the conditional probabilities under different states of RIFs.

Subsequently, the conditional probabilities related to variable V are represented by the set $t=\left\{t_{\text{1}},t_{\text{2}},\ldots ,t_{m}\right\}$, where the initial value $t_{\text{0}}$ is assigned based on expert estimation or relevant experience. To minimize the divergence between expert-elicited prior estimations y and empirical observations x, the Euclidean distance is adopted as the objective function:


$$\begin{array}{c} \begin{array}{c} d(x,y)=\sum_{t}{\left(x_{i}-y_{i}\right)}^{\text{2}} \end{array} \end{array}$$ (14)

The gradient descent algorithm is then applied to find the optimal conditional probability set t by minimizing d(x,y) through the following steps:

Step 1: Calculate the gradient of the objective function d(x,y) with respect to the conditional probability set t.
Step 2: Apply an update displacement Δt to $t_{\text{0}}$ in the opposite direction of the computed gradient.
Step 3: Iteratively repeat the process until the gradient converges to zero.
Step 4: Record the final optimized values of set t as the conditional probability tables (CPTs) in the BN model.

### 3.5 Model validation

#### 3.5.1 Confusion matrix.

The confusion matrix serves as a precise tool for evaluating the performance of predictive models [59,60]. In this study, the predicted railway operational accident risks are aligned with the confusion matrix to assess the accuracy of the developed predictive model. Table 2 provides an example of a confusion matrix for a three-class problem. The columns and rows of the matrix represent the predicted classes and actual classes, respectively. For example, $A_{\text{11}}$ denotes the number of samples where the actual class is 1 and the predicted result is also class 1, while $A_{\text{12}}$ represents the number of samples where the actual class is 1 but the prediction is class 2.

**Table 2:** Example of confusion matrix for three categories.

		Predicted class		
		Class 1	Class 2	Class 3
Actual class	Class 1	*A*_11_	*A*_12_	*A*_13_
	Class 2	*A*_21_	*A*_22_	*A*_23_
	Class 3	*A*_31_	*A*_32_	*A*_33_

To quantitatively substantiate the effective of the developed model, we propose an accuracy metric, which is typically calculated by using Eq. 15. A higher value indicates superior predictive performance.


$$\begin{array}{c} \begin{array}{c} Accuracy=\frac{\sum_{i=\text{1}}^{n}{TN}_{i}}{\sum_{i=\text{1}}^{n}{TN}_{i}+\sum_{i=\text{1}}^{n}\sum_{j=\text{1}}^{n}{FN}_{ij}} \end{array} \end{array}$$ (15)

where ${TN}_{i}$ represents the number of samples that the model predicts as the i−th state when the actual risk state is the i−th state, and FN represents the number of samples that the model incorrectly predicts as the j−th state when the actual risk state is the i−th state.

#### 3.5.2 Axioms test.

Once the BN model is constructed, it satisfies the following three axioms to validate the effectiveness of the proposed model:

Axiom 1: A small increase or decrease in the subjective prior probability of any parent node must necessarily result in a corresponding relative increase or decrease in the posterior probability of the child node.
Axiom 2: Given changes in the subjective probability distribution of each parent node, the magnitude of its influence on the child node value should remain consistent.
Axiom 3: The total magnitude of influence on the child node value resulting from the combined probability changes of all parent nodes should always be greater than the magnitude of influence exerted by any single parent node on its respective child node value.

#### 3.5.3 Real case test.

To strengthen the empirical validity of the proposed model, stochastic sampling from historical accident reports is performed to facilitate simulation test. By extracting pertinent RIFs from selected reports and mapping them onto the constructed BN, the congruence between simulated inferences and observed outcomes is scrutinized. This approach enables a granular appraisal of the model’s precision, ensuring that the proposed model remains grounded in operational reality.

### 3.6 Sensitivity analysis

#### 3.6.1 Mutual information.

Within the BN model, mutual information (MI) as a robust information-theoretic metric for quantifying systemic dependencies between stochastic variables. Its primary utility lies in providing a quantitative proxy for the entropic coupling and bidirectional influence characterizing the network nodes. By deriving MI, researchers can identify the key variables [61].

Furthermore, MI demonstrates exceptional adaptability to the non-linearities and multivariable interactions prevalent in railway systems. Through the examination of MI gradients, latent causal trajectories are uncovered, yielding critical insights that inform subsequent model optimization. The formula for calculating MI is as follows:


$$\begin{array}{c} \begin{array}{c} H\left(X_{\text{1}};X_{\text{2}}\right)=\sum_{x_{\text{1}}<X_{\text{1}}x_{\text{2}}<X_{\text{2}}}\;P\left(x_{\text{1}},x_{\text{2}}\right)\text{log}\left(\frac{P\left(x_{\text{1}},x_{\text{2}}\right)}{P\left(x_{\text{1}}\right)P\left(x_{\text{2}}\right)}\right) \end{array} \end{array}$$ (16)

where $X_{\text{1}}$ and $X_{\text{2}}$ represent two metrics in the BN, $P(x_{\text{1}},x_{\text{2}})$ denotes the joint probability of indicators $X_{\text{1}}$ and $X_{\text{2}}$, and $P(x_{\text{1}})$ and $P(x_{\text{2}})$ correspond to the marginal distribution probabilities of indicators $X_{\text{1}}$ and $X_{\text{2}}$, respectively.

#### 3.6.2 Weighted influence analysis.

While MI effectively quantifies stochastic associations between network variables, a high MI variable does not inherently signify significant influence [62]. To transcend the limitations of conventional sensitivity metrics, we adopt a weighted influence analysis method to achieve a more discriminatory appraisal of risk factor contributions. The specific steps of this method are as follows:

Step 1. Adjust the state of the target node risk to 100% to obtain the state distribution of each indicator under different accident severity levels.
Step 2. According to Eq. 15, determine the degree of influence of different states of the target node on each risk factor.

$$\begin{array}{c} \begin{array}{c} ID_{mn}=\sum_{s}\;\left(P_{mns}-P_{mn}\right)\times C_{s} \end{array} \end{array}$$ (17)

Step 3. For each RIF, a “critical failure state” is identified. This is defined as the state maximally predisposed to catastrophic system escalation, characterized by the maximum value among all potential variable states.
Step 4. The systemic impact induced by variations in each RIF is quantified by benchmarking $ID_{mn}$ against the worst-case state (represented as IDL).

To provide a holistic visualization of the risk propagation, two novel parameters-AOP and DV-are introduced. AOP represents the average probability of the worst occurrence of each risk factor under various risk states. The larger the value of AOP, the stronger the impact on the severity of higher risks, and more attention is needed. DV represents the difference between the highest and lowest probabilities of occurrence.

#### 3.6.3 Scenario analysis.

To rigorously probe the influence of diverse factors on the target node, this study implements scenario analysis. By modulating the operational configurations of these RIFs, multiple potential scenarios are constructed, facilitating the observation of the probabilistic response of the target node under different conditions [63]. This method enables systematic evaluation of the impact of various risk factors in different states on the probability and severity of accident occurrence. Eq. 18 is used to calculate the degree of influence of each node’s state on the target node.


$$\begin{array}{c} \begin{array}{c} P=\sum_{k=\text{1}}^{n}\;e_{k}\times f_{k} \end{array} \end{array}$$ (18)

where $e_{k}$ represents the probability distribution of the target node being in state k, while $f_{k}$ represents the fixed coefficient of the target node being in state k.

## 4 Case study

### 4.1 Data collection and processing

This study selects railway accidents in China from 2015 to 2024 as the research data. The data are sourced from publicly available official reports of the National Railway Administration, with a total of 168 accident reports collected. These reports cover various types of railway accidents, including derailments, collisions, overspeeding, and runaway trains. It is worth noting that, due to differences in reporting practices across years, some reports contain incomplete descriptions, making it impossible to extract risk-influencing factors. To ensure the reproducibility of this study, strict exclusion criteria were implemented during the report cleaning phase. If the following situations occur, the report will be discarded: (1) the accident report lacks clear causal conclusions approved by the investigating authority; (2) It contains missing values for over 50% of company management or risk check variables; (3) its descriptive records are insufficient to infer the classification status. Therefore, after excluding these incomplete reports, 143 accident reports are ultimately retained for analysis. Fig 5 illustrates the distribution of railway operational accidents based on severity. It is evident that the data exhibit an imbalanced structure.

**Fig 5:**
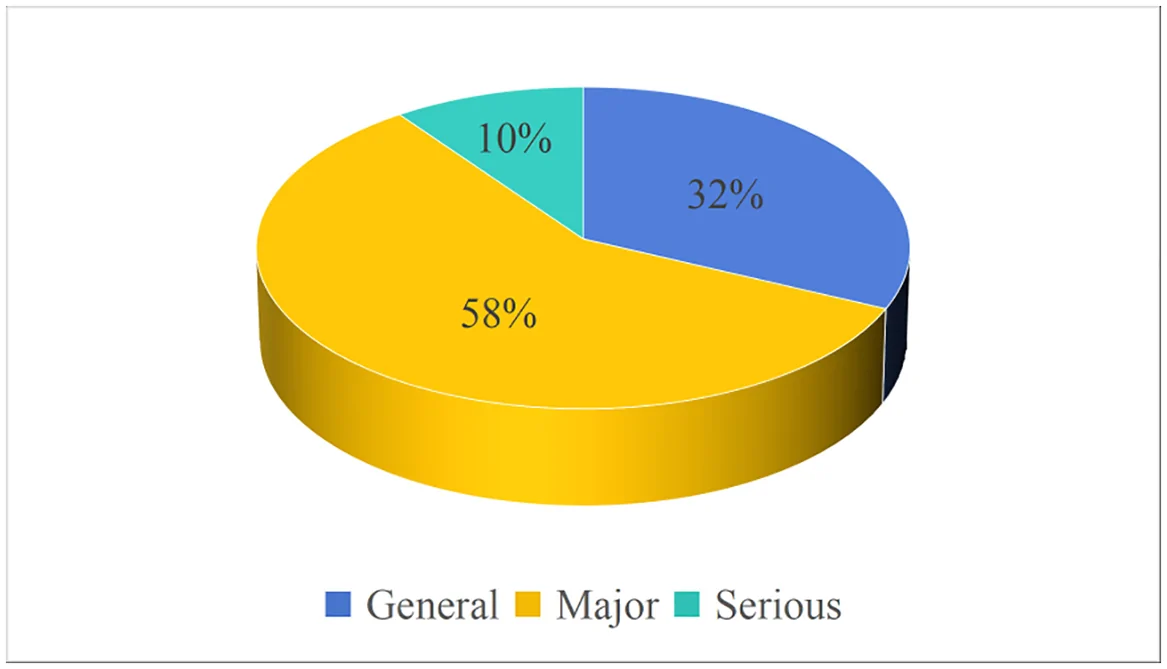
Distribution of railway accidents.

In addition, in order to strictly avoid data leakage and ensure the unbiasedness of model validation, the entire dataset of 143 accident reports was divided into an 80% training set (114 accident reports) and a 20% testing set (29 accident reports). The ADASYN algorithm was subsequently implemented only in the training set to address class imbalance, and was extended to 246 balanced training instances. In contrast, the 29 accident reports in the test set maintain their original, unaudited experience distribution as an objective evaluation benchmark.

As shown in Table 3, preliminary observations indicate that the assigned values directly correspond to the state configurations of the RIFs, providing a more straightforward way to describe the data distribution. First, the standard deviations of technical means, personnel fatigue, passenger behavior, and visibility are relatively high, suggesting significant deviations from their respective means. In addition, no missing data are found in the compiled dataset, which provides a solid foundation for applying the gradient descent algorithm. Table 4 presents the statistical analysis of the relevant variables after applying the ADASYN algorithm to balance the data. The results show that, following the implementation of ADASYN, changes occurred in the mean and standard deviation of the data, and the balance of the dataset was improved.

**Table 3:** Relevant variables and statistical data.

Variable	Number	Value	Max	Min	Mean	STD
Policy program	143	1;2	2	1	1.769	0.423
Supervisory system	143	1;2	2	1	1.510	0.502
Situation report	143	1;2	2	1	1.357	0.481
Technical regulations	143	1;2	2	1	1.790	0.409
Primary responsibility	143	1;2	2	1	1.510	0.502
Skill training	143	1;2	2	1	1.860	0.348
Work Standard	143	1;2	2	1	1.790	0.409
Management mechanism	143	1;2	2	1	1.357	0.481
Maintenance operation	143	1;2	2	1	1.615	0.488
Design quality	143	1;2	2	1	1.888	0.316
Technical measure	143	1;2	2	1	1.951	0.217
Equipment Management	143	1;2	2	1	1.846	0.362
Inspection mechanism	143	1;2	2	1	1.755	0.431
Risk judgment	143	1;2	2	1	1.531	0.501
Inspection form	143	1;2	2	1	1.776	0.418
Preventive measure	143	1;2	2	1	1.741	0.439
Personnel fatigue	143	1;2	2	1	1.937	0.244
Personnel communication	143	1;2	2	1	1.839	0.369
Personnel operation	143	1;2	2	1	1.517	0.501
Passenger behavior	143	1;2	2	1	1.972	0.165
Visibility	143	1;2	2	1	1.965	0.184
Time	143	1;2	2	1	1.930	0.256
Extreme weather	143	1;2;3	3	1	1.154	0.399
Equipment status	143	1;2	2	1	1.769	0.423
Track intrusion	143	1;2	2	1	1.706	0.654

**Table 4:** Relevant variables and statistical data after balancing.

Variable	Number	Value	Max	Min	Mean	STD
Policy program	246	1;2	2	1	1.667	0.472
Supervisory system	246	1;2	2	1	1.524	0.500
Situation report	246	1;2	2	1	1.366	0.483
Technical regulations	246	1;2	2	1	1.833	0.373
Primary responsibility	246	1;2	2	1	1.524	0.500
Skill training	246	1;2	2	1	1.841	0.366
Work Standard	246	1;2	2	1	1.833	0.343
Management mechanism	246	1;2	2	1	1.366	0.483
Maintenance operation	246	1;2	2	1	1.642	0.480
Design quality	246	1;2	2	1	1.919	0.274
Technical measure	246	1;2	2	1	1.943	0.232
Equipment management	246	1;2	2	1	1.882	0.323
Inspection mechanism	246	1;2	2	1	1.801	0.400
Risk judgment	246	1;2	2	1	1.520	0.501
Inspection form	246	1;2	2	1	1.752	0.433
Preventive measure	246	1;2	2	1	1.691	0.463
Personnel fatigue	246	1;2	2	1	1.963	0.188
Personnel communication	246	1;2	2	1	1.850	0.358
Personnel operation	246	1;2	2	1	1.512	0.501
Passenger behavior	246	1;2	2	1	1.951	0.216
Visibility	246	1;2	2	1	1.967	0.178
Time	246	1;2	2	1	1.927	0.261
Extreme weather	246	1;2;3	3	1	1.159	0.388
Equipment status	246	1;2	2	1	1.817	0.387
Track intrusion	246	1;2	2	1	1.720	0.450

In summary, the statistical comparison between Tables 3 and 4 indicates that the mean and standard deviation of all 25 RIFs remain consistent. ADASYN synthesized several instances on the local linear path between nearest neighbors without introducing abnormal data anomalies. In addition, to prevent model validation bias caused by synthetic data, the dataset of 143 accident cases was divided into an 80% training set and a 20% testing set. ADASYN is executed in the training set, while the test set retains its original imbalanced properties to ensure objective evaluation.

### 4.2 Identification of RIFs

Based on the collation and analysis of railway operational accident data, a risk assessment indicator system is constructed with accident risk as the target node. Taking into account the specific characteristics of railway operational accidents, this study applies the AcciMap to analyze the types of RIFs. For qualitative factors, state classifications are performed according to official standards; for quantitative factors, new states are assigned in combination with different levels. As a result, a railway operational accident risk assessment model comprising six key dimensions and 25 relevant factors is developed, as shown in Table 5.

**Table 5:** Description and status setting of RIFs.

Class	Variable	Description	State	State setting	Class	Variable	Description	State	State setting
Target	Risk	Degree of risk	General; Major; Serious	S1; S2; S3	Risk inspection	Inspection mechanism	Whether the risk inspection mechanism is complete	Yes; No	S1; S2
Government regulation	Policy program	Whether policy program is reasonable	Yes; No	S1; S2		Risk judgment	Whether the risk judgment methods are reasonable	Yes; No	S1; S2
	Supervisory system	Whether the supervisory system is reasonable	Yes; No	S1; S2		Inspection form	Whether the form of risk inspection is correct	Yes; No	S1; S2
	Situation report	Whether accident situation report is timely	Yes; No	S1; S2		Preventive measure	Whether the risk prevention measures are scientific	Yes; No	S1; S2
	Technical regulations	Whether the formulation of technical regulations is reasonable	Yes; No	S1; S2	Physical processes and actor activities	Personnel fatigue	Whether personnel perform operations within the prescribed time	Yes; No	S1; S2
Enterprise management	Primary responsibility	Whether the responsibility of management personnel is implemented	Yes; No	S1; S2		Personnel communication	Whether communication among personnel is effective	Yes; No	S1; S2
	Skill training	Whether the enterprise organizes training	Yes; No	S1; S2		Personnel operation	Whether personnel operations are standardized	Yes; No	S1; S2
	Work Standard	Whether work standards are reasonable	Yes; No	S1; S2		Passenger behavior	Whether passenger behavior is reasonable	Yes; No	S1; S2
	Management mechanism	Whether the enterprise management mechanism is complete	Yes; No	S1; S2	Equipment and surrounding	Visibility	Visibility distance	Good; Poor	S1; S2
Operation and maintenance management	Maintenance operation	Whether equipment maintenance operations are compliant	Yes; No	S1; S2		Time	Accident time	Day; Night	S1; S2
	Design quality	Whether design quality meets standards	Yes; No	S1; S2		Extreme weather	Weather conditions such as wind, rain, snow, etc.	General; Major; Serious	S1; S2; S3
	Technical measure	Whether maintenance technical means are advanced	Yes; No	S1; S2		Equipment status	Whether relevant equipment is in good condition	Yes; No	S1; S2
	Equipment management	Whether equipment management is reasonable	Yes; No	S1; S2		Track intrusion	Whether foreign objects are found on the track	Yes; No	S1; S2

It should be noted that strictly following the operation coding rules defined in Table 5, 25 RIFs are extracted qualitatively to quantitatively from the accident investigation report. To ensure coding reliability, all accident reports are independently cross coded by two researchers, and any ambiguity is resolved through consensus based on standard railway operating procedures. The complete structured dataset required to replicate all report results can be publicly accessed via https://github.com/gliu9572-create/Accident-data-set-of-China.git.

Factors related to government regulation include policy program, supervisory system, situation report, technical regulations. Factors related to enterprise management include primary responsibility, skill training, work standard, management mechanism. Factors related to operation and maintenance management include maintenance operation, design quality, technical measure, equipment management. Factors related to risk inspection include inspection mechanism, risk judgment, inspection form, preventive measure. Factors related to physical processes and actor activities include personnel fatigue, personnel communication, personnel operation, passenger behavior. Factors related to equipment and surrounding include visibility, time, extreme weather, equipment status, and track intrusion.

Under the guidance of the AcciMap framework, the risk propagation pathways and associated patterns across different levels were systematically analyzed, as illustrated in Fig 6.

**Fig 6:**
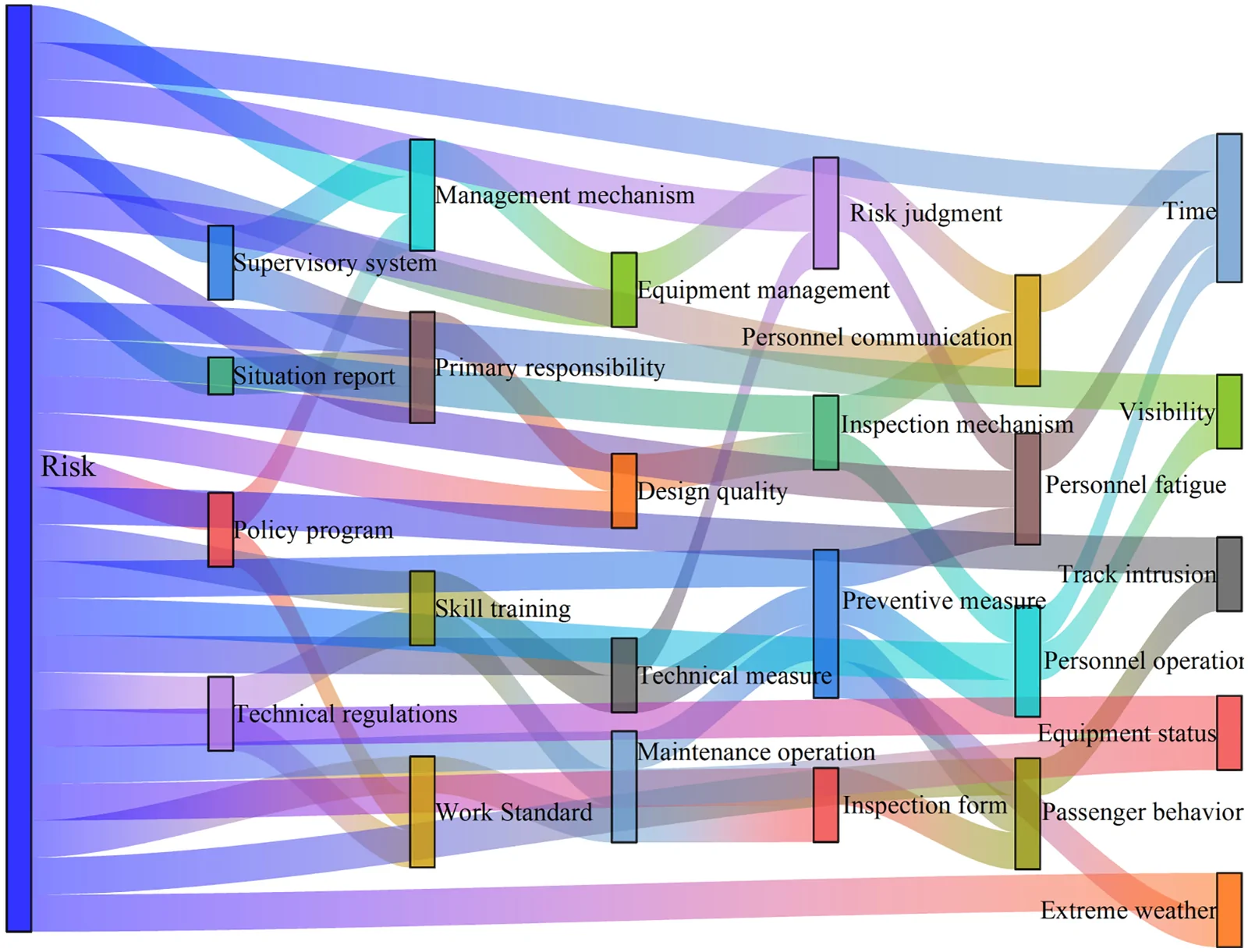
Structure diagram of AcciMap.

### 4.3 Results of railway operational accident risk analysis

In the Bayesian network (BN), prior probabilities for parent nodes were determined using the cloud model approach. Due to the absence of comprehensive empirical data for several risk factors, these probabilities were elicited primarily from domain experts, drawing on their professional judgment and accumulated experience.

This process benefited substantially from consultations with three highly qualified experts in railway operations and safety:

(1) A senior professor from Beijing Jiaotong University with expertise in operations management and risk analysis;
(2) A senior researcher with extensive experience in railway transportation safety; and
(3) A specialist in risk prediction, possessing in-depth knowledge of accident causation mechanisms.

Their collective input ensured robust and defensible quantification of subjective elements in the model.

#### 4.3.1 Results of the hesitant cloud model.

To handle nodes involving substantial subjective judgment, this study applied the hesitant cloud model. Parameters for these nodes were derived through a systematic evaluation informed by detailed interpretations of accident investigation reports.

For illustration, consider the risk factors associated with physical processes and operational activities. All such factors were mapped to a normalized probability scale [0, 1], with states defined as follows:

(1) For binary assessment: “good” corresponds to [0, 0.5] and “bad” to [0.5, 1].
(2) For ternary assessment: “good” corresponds to [0, 0.333], “medium” to [0.333, 0.667], and “bad” to [0.667, 1].

The general form of the cloud model characterizing the child nodes under each linguistic comment is given below.

The general expression of the cloud model for the comment “good” (L1) is:


$$\begin{array}{c} \begin{array}{c} \left\{ \begin{array}{cc} & Ex=\text{0}.\text{25}\\ & En=\frac{\text{1}}{\text{12}}\\ & He=\text{0}.\text{5} \end{array}\right.\; \end{array} \end{array}$$ (19)

The general expression of the cloud model for the comment “bad” (L2) is:


$$\begin{array}{c} \begin{array}{c} \left\{ \begin{array}{cc} & Ex=\text{0}.\text{75}\\ & En=\frac{\text{1}}{\text{12}}\\ & He=\text{0}.\text{5} \end{array}\right.\; \end{array} \end{array}$$ (20)

Subsequently, the analytic hierarchy process (AHP) is used to determine the weights of the three experts and the weights of each child node. The expert weights are set as shown in Table 6, and the child node weights are set as shown in Table 7.

**Table 6:** Weight table of three experts.

	Expert1	Expert2	Expert3
Personnel fatigue	0.3	0.4	0.3
Personnel communication	0.35	0.35	0.3
Personnel operation	0.2	0.45	0.35
Passenger behavior	0.4	0.3	0.3

**Table 7:** Weight table of child nodes.

	Personnel fatigue	Personnel communication	Personnel operation	Passenger behavior
Weight	0.25	0.30	0.25	0.2

The process of evaluating the results for physical processes and actor activities is illustrated in Fig 7. It is evident that the first four columns display the hesitant clouds of the three evaluators for the four child nodes: Expert 1 provides hesitant comments on “personnel fatigue,” “personnel operations,” and “passenger behavior”; Expert 2 provides hesitant comments on “personnel communication” and “passenger behavior”; and Expert 3 provides hesitant comments on “personnel fatigue” and “personnel communication.”

**Fig 7:**
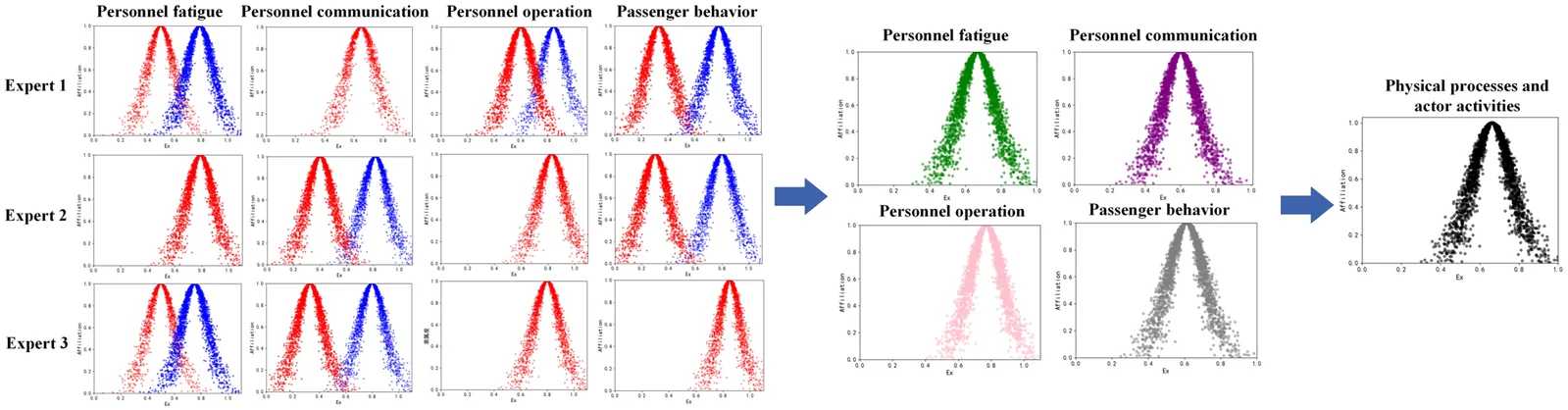
Flowchart of evaluating physical processes and actor activities through hesitation cloud model.

The fifth and sixth columns combine the average hesitant clouds from the three evaluators’ comments on these four risk factors, and the seventh column integrates the average hesitant clouds to obtain the comprehensive cloud for physical processes and operational activities. Then, cloud drops are generated, and the percentages of cloud drops falling within the intervals “Yes” [0, 0.5) and “No” [0.5, 1) are calculated, as shown in Table 8.

**Table 8:** Comprehensive cloud drops on intervals for physical processes and actor activities.

Comment	Yes	No
Percentage of cloud drops	0.2581	0.7419

As indicated in Table 8, physical processes and actor activities are assessed as “No” indicating that this factor is unfavorable to railway operational safety. This finding is consistent with the direct and indirect safety issues identified in the investigation reports as contributing to the accidents. For example, train drivers working extended hours without sufficient rest led to fatigue during driving; passengers ignored warning signs and staff dissuasion, resulting in crossing the tracks and colliding with train.

#### 4.3.2 Risk assessment results.

This study utilizes the Python environment and uses the ADASYN to transform imbalanced data into balanced data. Subsequently, the computational structure of the hesitant cloud model is used to obtain the prior probabilities of parent nodes for further calculation. Based on the balanced dataset, the gradient descent algorithm is employed to derive the conditional probability distributions of each RIF. The analysis results are shown in Fig 8.

**Fig 8:**
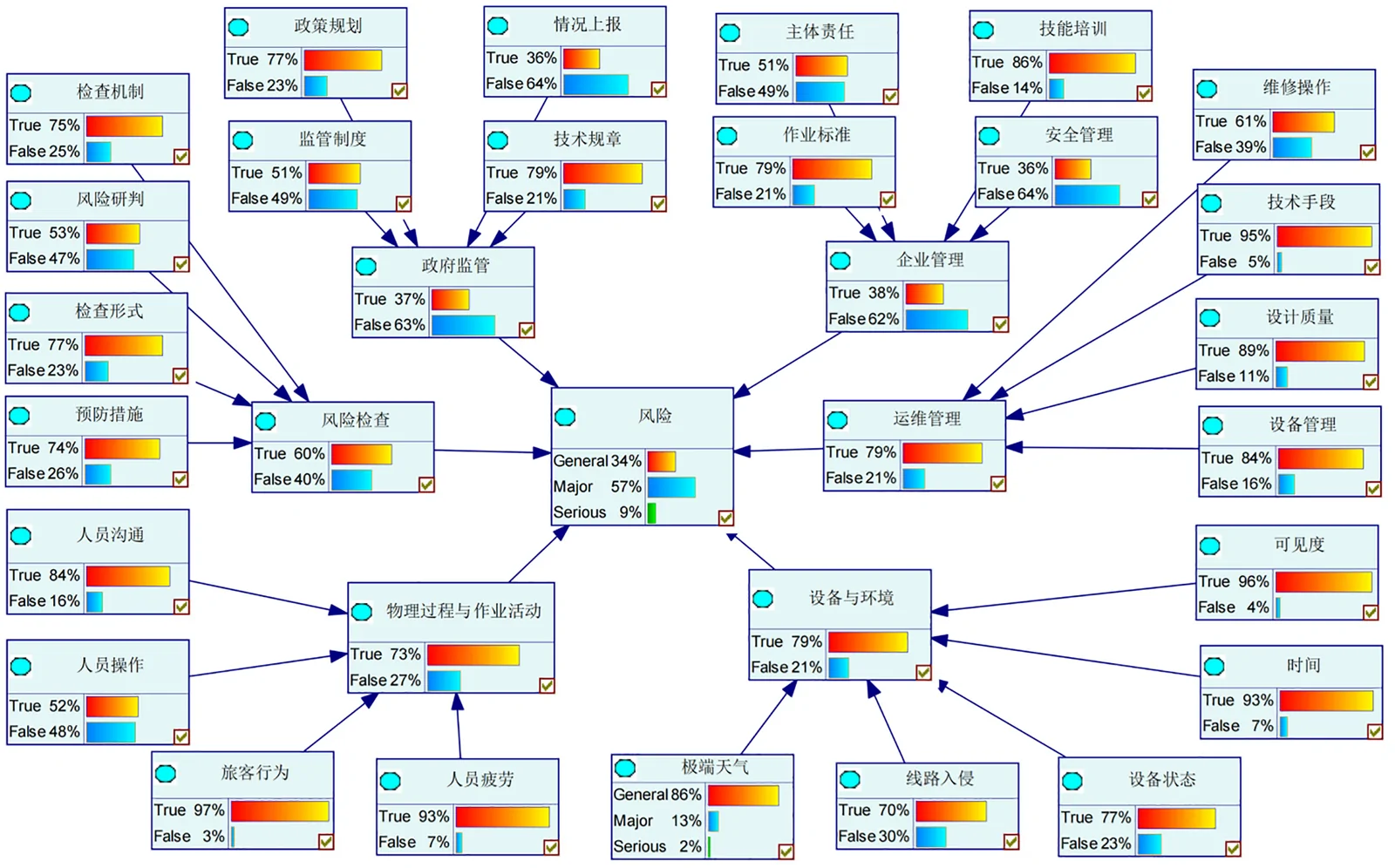
BN model for predicting railway operation accident risk.

Fig 8 presents the BN for railway operational accident risk. The posterior probabilities of the nodes highlight key insights into the distribution of risk severity across three categories: 34%, 57%, and 9%. In comparison, the distribution of risk severity categories in the initial dataset was recorded as 32%, 58%, and 10%. These findings demonstrate a significant consistency between the two sets of accident risk severity distribution probabilities, providing preliminary validation of the accuracy of the developed dynamic model.

### 4.4 Model validation

#### 4.4.1 Comparative evaluation with baseline models.

To verify the effectiveness of the proposed model, comparative experiments were conducted with four baseline models. The four baseline models are as follows: (1) Baseline model 1, represents BN without using the hesitant cloud model; (2) Baseline model 2, represented as BN without ADASYN; (3) Baseline model 3, represented by Random Forest (RF); (4) Baseline model 4, represented by support vector machine (SVM).

The evaluation indicators for model performance are accuracy, precision, F1-score, and recall. The performance indicators of all models are shown in Table 9. As shown in Table 9, the proposed model (AcciMap+Hesitant cloud+ADASYN-BN) achieved the highest accuracy and F1 score of 89.65% and 0.881, respectively.

**Table 9:** Performance comparison between the proposed model and baseline models.

Name	Model combination	Accuracy	Precision	Recall	F1-score
Proposed Model	Hesitant cloud+ADASYN-BN	89.66%	0.892	0.876	0.881
Baseline model 1	ADASYN-BN	79.31%	0.785	0.771	0.773
Baseline model 2	Hesitant cloud -BN	72.41%	0.746	0.648	0.669
Baseline model 3	RF (with ADASYN)	85.86%	0.851	0.842	0.844
Baseline model 4	SVM (with ADASYN)	82.41%	0.818	0.895	0.802

The accuracy of baseline model 1 significantly decreased to 79.31%, indicating that using the hesitant cloud model provides better prior probabilities than the standard arithmetic mean and reduces cognitive subjective noise. The recall of baseline model 2 sharply decreased to 0.648, indicating that in cases of serious class imbalance, the model tends to misclassify rare serious accidents into the major accident. The baseline model (RF) and baseline model 4 (SVM) achieved satisfactory accuracy of 85.86% and 82.41%, but RF and SVM as machine learning models, they have the “black box” characteristic, so they cannot perform causal inference and dynamic scene inference.

#### 4.4.2 Confusion matrix.

To evaluate the predictive performance of the developed BN model, this study utilized a confusion matrix. This matrix provides a structured overview of the model classification results by comparing the predicted risk categories with the actual observed conditions in the dataset, as shown in Table 10. Rows represent the predictions of the BN model, while columns reflect the true state of the accident case.

**Table 10:** Confusion matrix of BN.

		Predict class		
		General	Major	Serious
Actual class	General	13	1	0
	Major	1	9	0
	Serious	0	1	4

According to the results in Table 10, the model correctly predicted 13 general accidents, 9 major accidents, and 4 serious accidents, with an overall prediction accuracy of 89.65%. This performance confirms that the model provides consistent and reliable risk severity prediction without overfitting the training distribution.

#### 4.4.3 Axioms validation.

To verify the logical consistency and structural validity of the proposed BN model, axiomatic validation was conducted using six selected RIFs from the constructed railway operational accident risk indicator system. These RIFs include “policy program”, “skill training”, “maintenance operation”, “preventive measure”, “personnel operation”, “equipment status”, with risk severity as the target node. The validation procedure involved incrementally increasing the prior probabilities of each selected parent node by 10% while monitoring the resulting posterior probability of the target node. Results are illustrated in Table 11. As demonstrated in Table 11, increasing the prior probability of any individual parent node produces a corresponding directional increase in the posterior probability of severe risk, thereby satisfying Axiom 1.

**Table 11:** Result of Axiom testing.

Factor	Status	Degree of impact						Degree of risk		
								General	Major	Serious
Initial model								22.47%	52.83%	25.34%
Policy program	S2	+10%						20.82%	54.27%	24.91%
Skill training	S2	+10%	+10%					18.65%	56.42%	24.93%
Maintenance operation	S2	+10%	+10%	+10%				16.43%	55.36%	28.21%
Preventive measure	S2	+10%	+10%	+10%	+10%			15.57%	53.73%	30.70%
Personnel operation	S2	+10%	+10%	+10%	+10%	+10%		14.18%	50.54%	35.28%
Equipment status	S2	+10%	+10%	+10%	+10%	+10%	+10%	12.64%	46.10%	41.26%

Furthermore, when the prior probabilities of multiple parent nodes are varied simultaneously, the posterior probability of severe risk follows a similar directional trajectory, confirming Axiom 2. Notably, the posterior probability of severe risk rose from an initial 25.34% to 41.26%. This increase is less than the sum of the individual effects from the parent node perturbations, indicating that the model appropriately captures non-additive interactions among risk factors, thereby validating Axiom 3.

#### 4.4.4 Real case validation.

To further validate the effectiveness of the developed BN model, this study randomly selected one real case from the compiled database for examination. On March 30, 2020, a railway operational accident occurred on the Yongxing section of the Beijing-Guangzhou Line, resulting in 1 death, 127 injuries, and economic losses of 22.35 million yuan. According to the accident classification standard, this incident was classified as a major railway traffic accident. According to the accident report records, the parameters of the 25 RIFs are shown in Table 12. It show that information regarding maintenance operation, design quality, technical measure, and equipment management was missing in the accident record and thus treated as “unknown—no locked evidence in Fig 9.”

**Table 12:** Accident details.

Factor	Status	Factor	Status
Risk	Serious	Inspection mechanism	No
Policy program	Yes	Risk judgment	No
Supervisory system	Yes	Inspection form	Yes
Situation report	No	Preventive measure	No
Technical regulations	Yes	Personnel fatigue	Yes
Primary responsibility	Yes	Personnel communication	No
Skill training	Yes	Personnel operation	Yes
Work Standard	Yes	Passenger behavior	Yes
Management mechanism	No	Visibility	Yes
Maintenance operation	Yes	Time	Day
Design quality	Yes	Extreme weather	Heavy rain
Technical measure	Yes	Equipment status	Yes
Equipment management	Yes	Track intrusion	No

**Fig 9:**
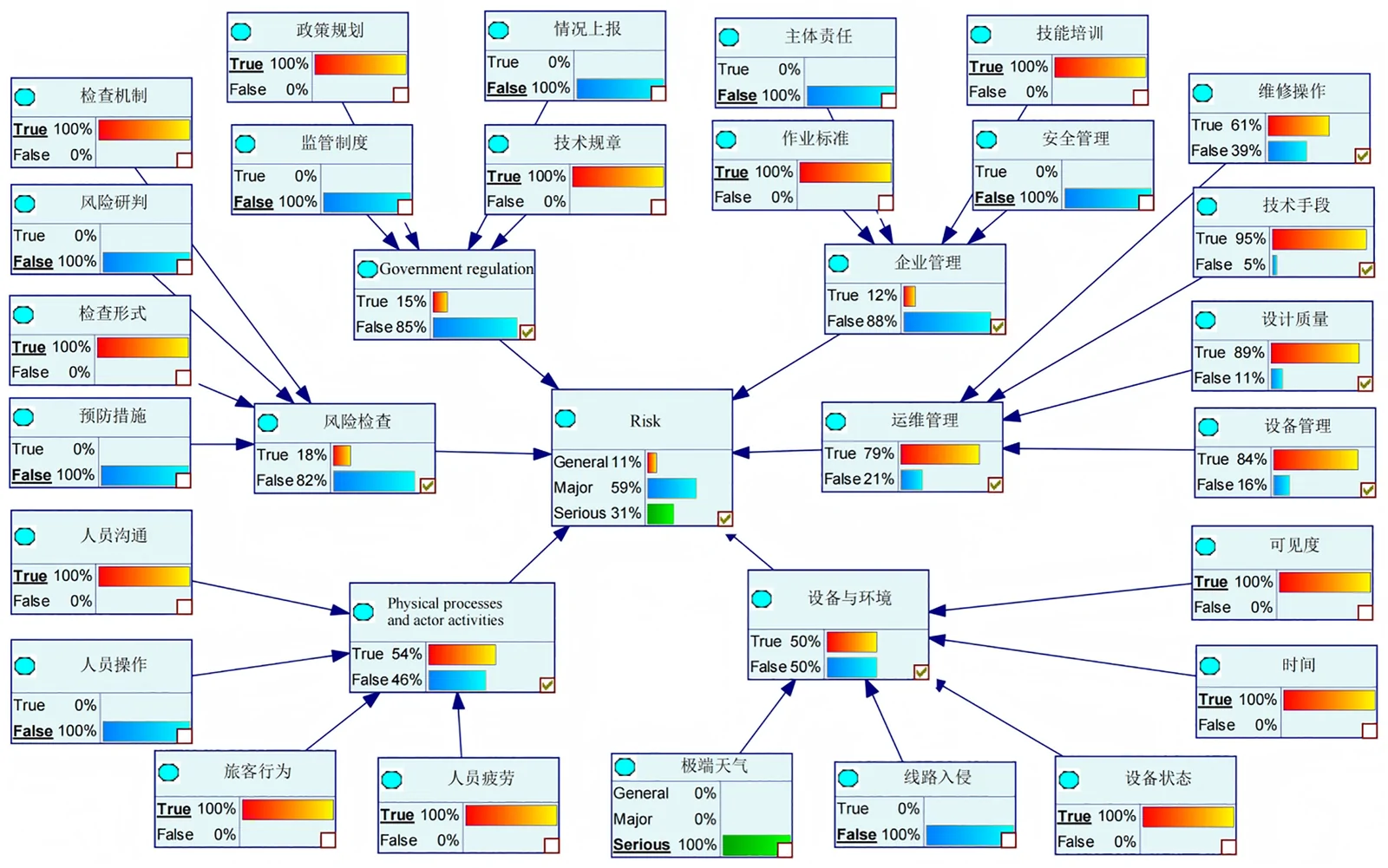
Real case verification.

Fig 8 shows the railway operation accident prediction network model, which reflects the overall probability relationship obtained from historical accident reports. To verify the actual effectiveness of the model, Table 12 summarizes the status of 25 RIFs extracted from official accident investigation reports. Based on this, the factors clearly recorded in the investigation report are identified as factual evidence in the network. For example, extreme weather status is Heavy rain, situation report status is No, etc. Meanwhile, for RIFs that were not recorded in the accident report, such as maintenance operation, design quality, technical measure, and equipment management, the accident was not involved. Therefore, the probabilities of these RIFs maintain the probability distribution of the proposed model, as shown in Fig 8.

After inputting the evidence of specific cases into the model, the updated posterior probability of major or higher-level accidents reaches 90%, with major accidents accounting for 59% and serious accidents accounting for 31%, as shown in Fig 9. This result is highly consistent with the actual classification of accidents, further demonstrating the effectiveness of the proposed model.

### 4.5 Sensitivity analysis

#### 4.5.1 Parameter analysis of hyper-entropy k.

In the hesitant cloud model, the hyper entropy parameter He=k in Eq. (10) controls the randomness and dispersion of cloud droplets, reflecting the uncertainty of entropy. To confirm that the selection of k=0.5 does not introduce subjective bias or instability, sensitivity analysis is conducted on parameter k, with k=0.3, 0.4, 0.5, 0.6, 0.7. We examine its impact on both the posterior risk probabilities in the real case and the accuracy of model. The results are shown in Fig 10. The results indicate that the change in k value has caused negligible fluctuations in risk classification. In addition, the accuracy of the model under different k values are verified, and the results remain basically stable. This confirms that the proposed model has excellent robustness to hyperentropic perturbations. Therefore, taking k=0.5 is reliable.

**Fig 10:**
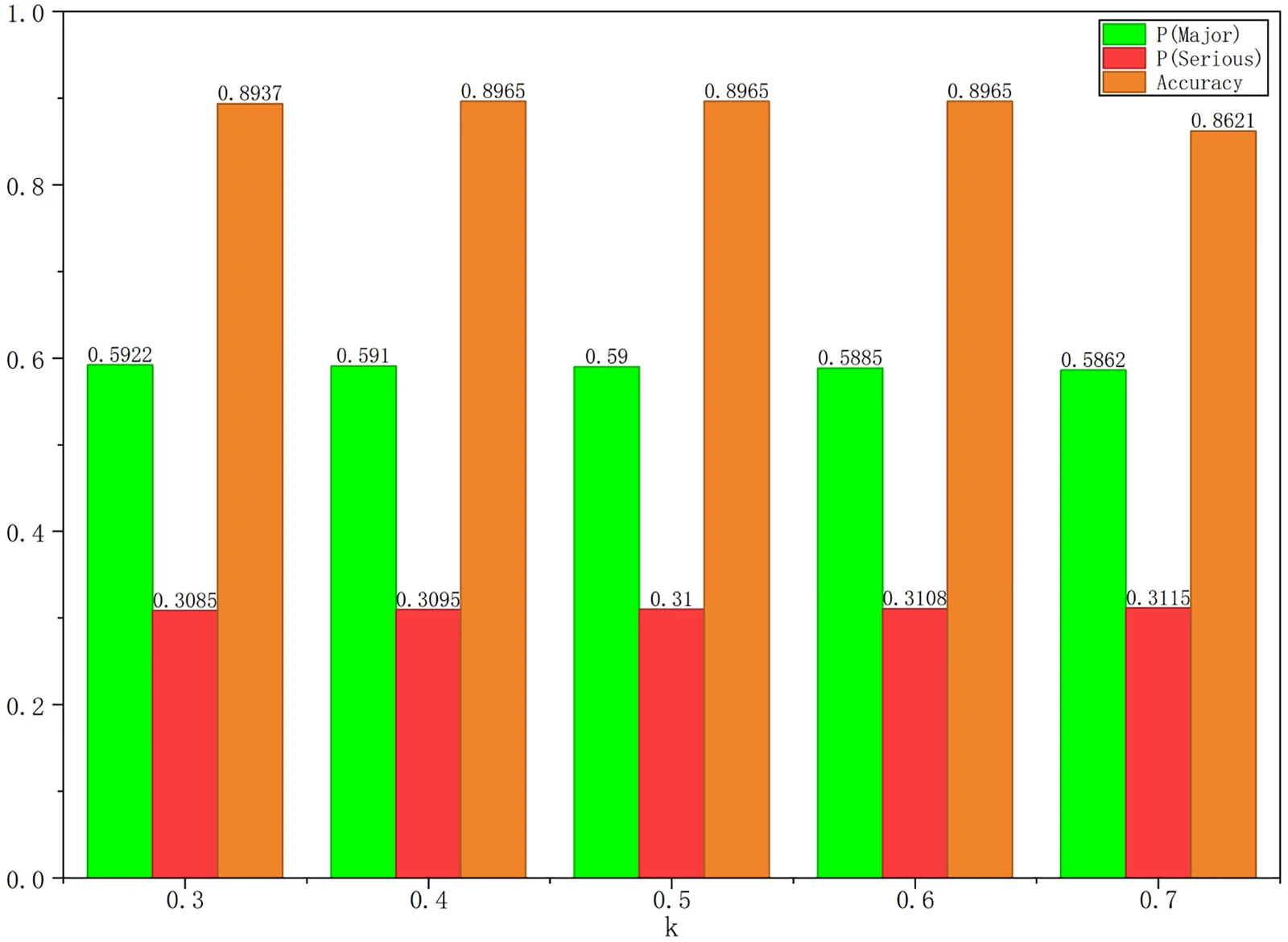
The influence of different parameters *k* on risk classification and model accuracy.

#### 4.5.2 Mutual information analysis.

MI is a widely adopted metric in railway operational accident risk modeling for quantifying the dependency and shared information between pairs of variables. A higher MI value between a parent node and the target node indicates stronger influence and greater informational contribution, which supports feature prioritization, model interpretation, and identification of dominant risk drivers.

In this study, “risk severity” served as the target node. The mutual information between each parent node and the target was computed according to Eq. 16. The results identified “extreme weather” as the most influential factor, with an MI value of 0.4128, “track intrusion”, “equipment status”, “personnel operation”, and “risk judgment”, with MI values of 0.2186, 0.1564, 0.1023, and 0.0897, respectively. Fig 11 displays the top ten risk-influencing factors ranked by MI value. These nodes are considered to have significant impact on risk severity and are therefore identified as critical RIFs.

**Fig 11:**
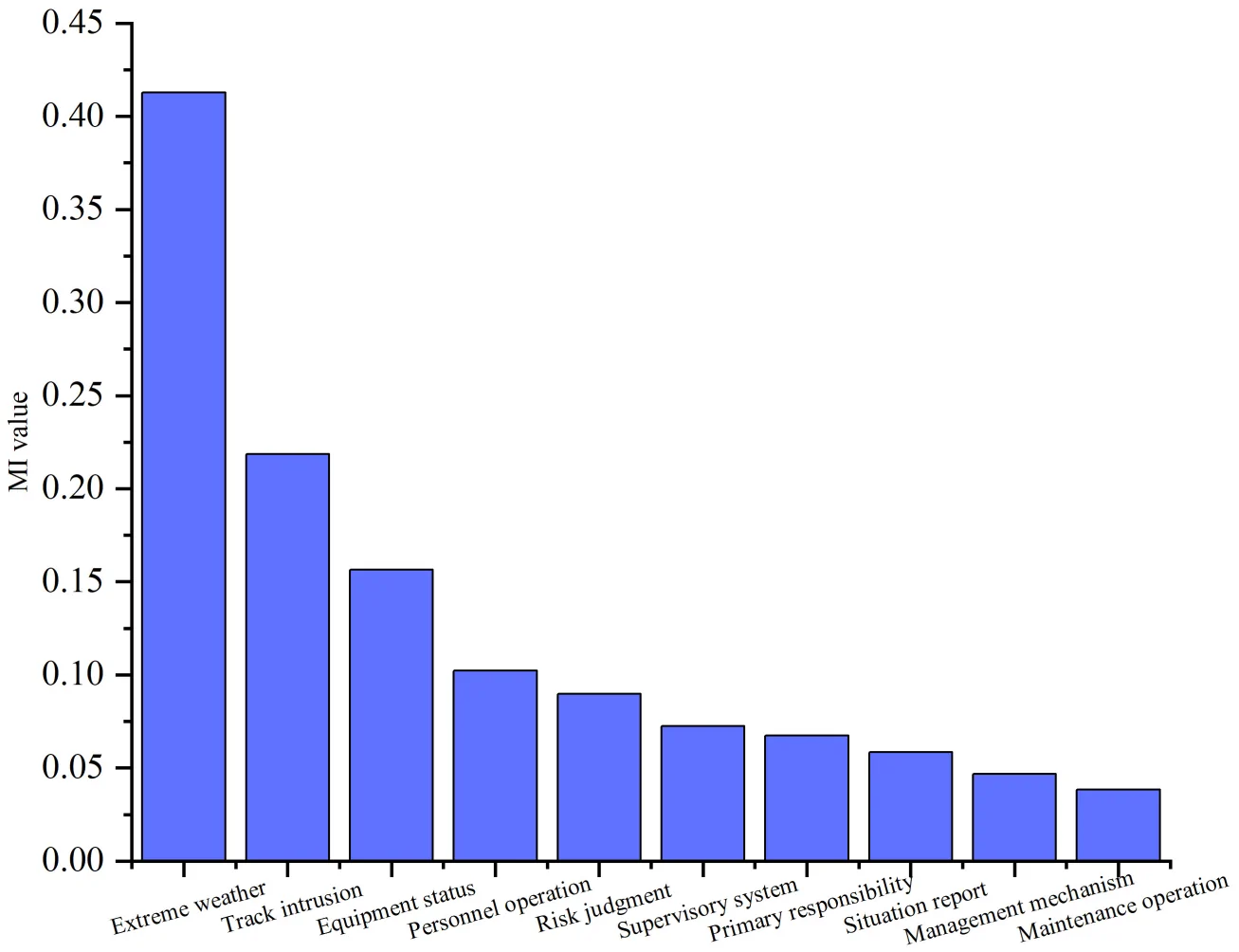
MI value of RIFs.

#### 4.5.3 Weighted influence analysis.

Given the substantial interdependencies among RIFs, relying on a single unidirectional measure may fail to fully capture the composite influence of one factor on another. To address this limitation, the weighted influence analysis is used and calculated using Eq. 17, with $C_{\text{1}}=\text{1},C_{\text{2}}=\text{10},C_{\text{3}}=\text{100}$. The results are presented in Table 13. These indicators are classified into different risk levels based on their AOP values: indicators with AOP values between 50% and 100% are categorized as “high impact,” those between 20% and 50% as “medium impact,” and those between 0% and 20% as “low impact.”

**Table 13:** The impact of different RIFs on the risk performance of severity risk.

Factor	Status	IDL	AOP	DV	Impact class
Management mechanism	No	7.46	72.85%	16.25%	High impact
Situation report	No	3.40	70.73%	17.28%	
Track intrusion	No	15.25	58.46%	30.49%	
Personnel operation	No	17.12	53.78%	32.93%	
Risk judgement	No	4.94	53.41%	13.41%	
Extreme weather	Serious	10.98	51.22%	12.44%	
Primary responsibility	No	5.82	46.25%	18.29%	Medium impact
Supervisory system	No	4.39	44.31%	20.68%	
Maintenance operation	No	9.55	36.99%	21.95%	
Preventive measure	No	11.41	35.77%	29.27%	
Passenger behavior	No	5.48	34.76%	9.76%	
Personnel communication	No	5.59	33.26%	14.53%	
Policy program	No	63.00	31.33%	65.80%	
Equipment status	No	4.94	29.92%	15.85%	
Personnel fatigue	No	8.89	27.35%	18.29%	
Work standard	No	15.37	21.92%	20.73%	
Inspection mechanism	No	9.87	20.95%	21.95%	
Visibility	Poor	12.29	16.50%	19.51%	Low impact
Skill training	No	8.56	14.63%	18.29%	
Technical measure	No	2.96	14.07%	6.50%	
Inspection form	No	9.03	12.96%	15.85%	
Technical regulation	No	12.18	12.76%	20.73%	
Equipment management	No	10.98	11.79%	19.51%	
Design quality	No	8.78	11.38%	14.63%	
Time	Night	14.82	9.76%	25.61%	

The findings of this study provide valuable insights for the prevention of railway operational accidents. In the high impact category, both “track intrusion” and “extreme weather” belong to the equipment and surrounding, underscoring the critical importance of these factors in railway operational accidents. This highlights the need for relevant authorities to apply advanced technological methods for intelligent management. Notably, extreme weather exhibits the highest MI value among all factors, at 0.4128. Therefore, in the context of equipment and surrounding, close attention must be paid to changes in extreme weather conditions, with timely and effective measures taken to mitigate their impact on railway operational safety. Furthermore, additional analysis reveals that “situation report”, “management mechanism”, “risk judgment” and “personnel operation” fall into the high-impact category, all of which have relatively high mutual information values. Consequently, it is necessary to focus particular attention on these factors to minimize their influence on railway operational accident risk.

In the medium impact category, “personnel communication”, “personnel fatigue”, and “passenger behavior” all belong to the physical processes and actor activities domain, indicating that personnel management cannot be overlooked. Notably, “policy program” exhibits the highest IDL and DV values among all factors, at 63.00% and 65.80%, respectively. This phenomenon suggests that “policy program” has a significant influence on the uncertainty of railway operational accident risk. Therefore, continuous monitoring and dynamic adjustment of policy program are required to better align it with railway operations. In addition, “maintenance operation”, “prevention measure”, “work standard”, and “inspection mechanism” show relatively high DV values, indicating that these factors exert considerable influence on the uncertainty of railway operational accident risk. This underscores the urgent need to strengthen preventive measures and response strategies targeting these influential factors.

Finally, factors such as “visibility”, “skill training”, “technical measure”, and “inspection form” belong to the low impact category. However, this does not imply that these factors should be neglected. On the contrary, it indicates that current management of these risk factors is effective, and continued attention to these areas is necessary to further reduce their contribution to railway operational accident risk. An interesting finding is that “time” has the smallest AOP value among all RIFs, suggesting that nighttime conditions have a relatively small impact on risk severity.

In summary, from an overall perspective, factors at the government regulation and enterprise management levels are primarily concentrated in the medium impact and low impact. This demonstrates that substantial efforts have already been made by government and enterprises in preventing railway operational accidents. Risk factors at the operation and maintenance management and risk inspection levels exhibit diverse distributions, reflecting elevated standards for railway operational conditions and pre-departure train inspections. Risk factors at the physical processes and actor activities level and the equipment and surrounding level are mainly concentrated in the high impact and medium impact, indicating the necessity to strengthen management of personnel, equipment, and environmental factors.

#### 4.5.4 Simulation scenario analysis.

Scenario simulation analysis is conducted to investigate the conditional impact of individual RIF states on accident risk severity under specific conditions. This approach enables railway safety managers to evaluate potential risk pathways. The top ten ranked by MI value are regarded as key RIFs. The simulation employs fixed weighting coefficients $f_{k}=\text{1},f_{\text{2}}=\text{10},f_{\text{3}}=\text{100}$, Using Eq. 18, the influence value of each state on the posterior probability of severe risk is calculated. The results are summarized in Table 14.

**Table 14:** Analysis results of risk influencing factors under different scenarios.

Factor	Status	Setting	Risk probability			Risk value
			General	Major	Serious	
Extreme weather	S1	100%	86.70%	11.30%	2.00%	3.99
	S2	100%	24.30%	67.80%	7.90%	14.92
	S3	100%	11.30%	38.70%	50.00%	53.98
Track intrusion	S1	100%	84.20%	12.80%	3.00%	5.12
	S2	100%	14.70%	45.30%	40.00%	44.67
Equipment status	S1	100%	85.40%	8.60%	6.00%	7.71
	S2	100%	12.70%	57.10%	30.20%	36.03
Personnel operation	S1	100%	82.70%	13.50%	3.80%	5.97
	S2	100%	15.30%	58.20%	26.50%	32.47
Risk judgement	S1	100%	84.20%	9.70%	6.10%	7.91
	S2	100%	13.50%	63.50%	23.00%	29.48
Supervision system	S1	100%	82.90%	13.70%	3.40%	5.59
	S2	100%	16.70%	60.80%	22.50%	28.74
Primary responsibility	S1	100%	87.40%	8.70%	3.90%	5.64
	S2	100%	21.60%	64.80%	13.60%	20.29
Situation report	S1	100%	88.20%	10.30%	1.50%	3.41
	S2	100%	17.40%	58.60%	24.00%	30.03
Management mechanism	S1	100%	82.60%	11.70%	5.70%	7.69
	S2	100%	24.70%	65.30%	10.00%	16.77
Maintenance operation	S1	100%	80.40%	12.60%	7.00%	9.06
	S2	100%	23.50%	62.80%	13.70%	20.21

Among all RIFs, the state “S3” of extreme weather exerts the most significant influence on the target node, reaching 53.98. This is followed by the state “S2” of line intrusion, with an influence value of 44.67. Therefore, railway operational safety managers should pay greater attention to these aspects and take timely measures to prevent train operations under such conditions.

## 5 Discussion and implications

Considering the multiple factors that collectively contribute to different risk levels in railway operations, this study adopts the AcciMap theory to construct an indicator system, thereby developing a multi-level risk assessment framework. Specifically, the proposed risk assessment framework incorporates the ADASYN algorithm to effectively mitigate class imbalance in the accident dataset, thereby improving model robustness and predictive performance on minority classes. Secondly, a core innovation lies in the development of the hesitant cloud model, which combines cloud model with hesitant fuzzy sets to better represent and process the inherent uncertainty, vagueness, and diversity present in expert judgments. In railway risk assessment, expert elicitation is frequently characterized by hesitation, inconsistency, and varying levels of confidence. By incorporating the hesitation degree, the proposed model enables more faithful encoding of these nuances and facilitates the meaningful aggregation of judgments from multiple experts into a coherent probabilistic distribution.

This aggregation strategy not only enhances the overall accuracy and stability of the risk estimates but also substantially reduces the disproportionate impact that any single expert’s opinion might otherwise exert on the final results. Consequently, the framework achieves greater comprehensiveness, objectivity, and reliability in quantifying and prioritizing railway operational accident risks.

Consequently, by integrating the hesitant cloud model with the BN, this study constructs an improved Bayesian network model that better accommodates epistemic uncertainty and expert hesitation in railway operational risk assessment. The incorporation of the hesitant cloud model markedly improves the model’s flexibility and responsiveness to evolving conditions, enabling more refined quantification of dynamic interdependencies among RIFs. The combination of these two approaches enables real-time and dynamic assessment of RIFs, providing decision-makers with timely and accurate insights into risk severity, thereby facilitating more informed and proactive risk management decisions.

The framework proposed in this study effectively addresses the challenge of imbalanced data across different risk severity levels, demonstrates superior capability in integrating diverse data formats in uncertain environments, and reduces subjectivity stemming from different experts. Model validation results confirm that the proposed model exhibits higher accuracy. It is worth noting that the Bayesian network framework proposed in this study is different from machine learning classifiers such as Random Forest or XGBoost in that it has causal interpretability. Based on a multi-level AcciMap hierarchical structure, it allows railway managers to track how organizational and technical failures propagate to proactive operational risks and perform quantitative diagnostic reasoning.

These findings have practical significance for railway operations, and the quantitative results of this study provide unique insights. (1) According to the weighted impact analysis results in Table 11, factors such as track intrusion, personnel operations, and risk assessment are classified as high impact layers. Therefore, railway management personnel should prioritize the inspection of high impact factors, strengthen protective measures around railway operations, focus on improving the abilities of operators, and actively promote the use of intelligent equipment. (2) According to the sensitivity analysis in Fig 10, extreme weather exhibits the highest mutual information and generates the most severe risk escalation in scenario simulations. Therefore, railway companies should establish a scientific meteorological warning mechanism to link real-time weather monitoring data with railway operations. When extreme weather conditions approach the safety threshold, issue timely warnings and provide scientific emergency response plans to avoid train derailment or collision accidents. (3) The high AOP value of management mechanisms and situation reports emphasizes the decisive role of organizational and communication efficiency in reducing the severity of accidents. Railway companies should continuously improve their management mechanisms, break the dilemma of cross disciplinary communication, and provide effective information for railway decision-makers. (4) By using the AcciMap framework to draw causal paths, railway companies can improve emergency responders for high probability accident scenarios. For example, when foreign objects are detected invading the track, multi departmental emergency protocols can be executed simultaneously to reduce response delays and prevent secondary catastrophic hazards.

## 6 Conclusions

This study conducts an in-depth investigation into a new framework for railway operational accident risk assessment. Firstly, to comprehensively evaluate different severity levels of railway accidents, the AcciMap theory is adopted to establish a comprehensive risk indicator framework. Secondly, to enhance data effectiveness, the ADASYN algorithm is utilized to address the data imbalance problem, enabling deeper exploration of associations among key RIFs. Thirdly, a data-driven BN model is constructed, and, in response to the characteristics of unstructured data provided by multiple experts for network nodes, the hesitant cloud model is innovatively proposed to optimize the BN, thereby accurately integrating subjective judgments from multiple experts. Finally, various validation methods are applied to demonstrate the superior performance of the proposed model. In addition, mutual information, weighted influence and simulation scenario are used to conduct sensitivity analysis on the proposed model, thereby identifying the critical RIFs. The findings of this study hold significant implications for the prevention of railway operational accidents. The conclusions of this paper are as follows:

(1) Through MI value calculation, the primary RIFs of railway operational accident are identified as “extreme weather,” “track intrusion,” “equipment status,” “personnel operation,” and “risk judgment.” Among these, the MI value of “extreme weather” is notably higher than that of other factors, underscoring its critical importance in railway safety management.
(2) The results of weighted influence analysis reveal that “equipment and surrounding” exerts a substantial influence on railway operational risk management. However, the related factors “visibility” and “time” fall within the low-impact range, indicating relatively minor influence on accident risk severity. This may be attributed to the heightened concentration and cautious operation of train drivers under low-visibility or nighttime conditions, which reduces the likelihood of accidents. In contrast, “management mechanism” and “situation report” exhibit relatively high AOP values, confirming that management factors have a greater impact on railway operational accident risk.
(3) Real case validation demonstrates that the proposed model achieves a predictive accuracy of 89.65%, enabling accurate prediction of railway accident risk severity and providing a reliable basis for railway operational accident prevention.

Although the risk assessment framework proposed in this study has been validated through confusion matrix and axiomatic testing methods, it still has certain limitations. For instance, the number of collected railway accident reports is relatively limited. Future research will incorporate more diverse datasets to build a more comprehensive railway operational accident database. Furthermore, designing reasonable and advanced machine learning methods to better balance imbalanced and heterogeneous datasets, such as class weighting, SMOTE-NC, hierarchical resampling and bayesian smoothing to reduce unobserved heterogeneity between RIFs deserve further attention. Meanwhile, comparative testing will be explored for different combination models to further investigate the performance of the models. In future work, we plan to expand this framework into a multidimensional dynamic railway operation risk assessment framework by integrating continuous temporal dynamics and spatial geographic attributes. This will better explain the impact of complex factors on railway operation risks and provide a more comprehensive description of the dynamic risk evolution in railway operations.
